# An Integrated Nomogram Combining Deep Learning and Radiomics for Predicting Malignancy of Pulmonary Nodules Using CT‐Derived Nodules and Adipose Tissue: A Multicenter Study

**DOI:** 10.1002/cam4.70372

**Published:** 2024-11-04

**Authors:** Shidi Miao, Qifan Xuan, Hanbing Xie, Yuyang Jiang, Mengzhuo Sun, Wenjuan Huang, Jing Li, Hongzhuo Qi, Ao Li, Qiujun Wang, Zengyao Liu, Ruitao Wang

**Affiliations:** ^1^ School of Computer Science and Technology Harbin University of Science and Technology Harbin China; ^2^ Department of Internal Medicine Harbin Medical University Cancer Hospital, Harbin Medical University Harbin China; ^3^ Department of Geriatrics The Second Affiliated Hospital, Harbin Medical University Harbin China; ^4^ Department of General Practice The Second Affiliated Hospital, Harbin Medical University Harbin China; ^5^ Department of Interventional Medicine The First Affiliated Hospital, Harbin Medical University Harbin China

**Keywords:** adipose tissue, computed tomography, deep learning, multicenter, multimodal, nomogram, pulmonary nodules, radiomics

## Abstract

**Background:**

Correctly distinguishing between benign and malignant pulmonary nodules can avoid unnecessary invasive procedures. This study aimed to construct a deep learning radiomics clinical nomogram (DLRCN) for predicting malignancy of pulmonary nodules.

**Methods:**

One thousand and ninety‐eight patients with 6–30 mm pulmonary nodules who received histopathologic diagnosis at 3 centers were included and divided into a primary cohort (PC), an internal test cohort (I‐T), and two external test cohorts (E‐T1, E‐T2). The DLRCN was built by integrating adipose tissue radiomics features, intranodular and perinodular deep learning features, and clinical characteristics for diagnosing malignancy of pulmonary nodules. The least absolute shrinkage and selection operator (LASSO) was used for feature selection. The performance of DLRCN was assessed with respect to its calibration curve, area under the curve (AUC), and decision curve analysis (DCA). Furthermore, we compared it with three radiologists. The net reclassification improvement (NRI), integrated discrimination improvement (IDI), and subgroup analysis were also taken into account.

**Results:**

The incorporation of adipose tissue radiomics features led to significant NRI and IDI (NRI = 1.028, *p* < 0.05, IDI = 0.137, *p* < 0.05). In the I‐T, E‐T1, and E‐T2, the AUCs of DLRCN were 0.946 (95% CI: 0.936, 0.955), 0.948 (95% CI: 0.933, 0.963) and 0.962 (95% CI: 0.945, 0.979), The calibration curve revealed good predictive accuracy between the actual probability and predicted probability (*p* > 0.05). DCA showed that the DLRCN was clinically useful. Under equal specificity, the sensitivity of DLRCN increased by 8.6% compared to radiologist assessments. The subgroup analysis conducted on adipose tissue radiomics features further demonstrated their supplementary value in determining the malignancy of pulmonary nodules.

**Conclusion:**

The DLRCN demonstrated good performance in predicting the malignancy of pulmonary nodules, which was comparable to radiologist assessments. The adipose tissue radiomics features have notably enhanced the performance of DLRCN.

## Graphical abstract

Development of a nomogram that combines intranodular and perinodular region, adipose tissue, and clinical data, improved classification accuracy with the AUC of 0.946, 0.948, and 0.962 in internal and external test cohorts. The performance of nomogram was comparable to radiologist assessments, under equal specificity, the average sensitivity of nomogram increased by 8.6% compared to radiologists. Integration of adipose tissue provides enhanced information to distinguish between benign and malignant nodules, both NRI and IDI showed significant improvement (*p* < 0.05).

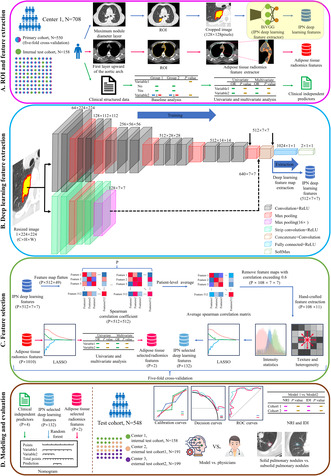

## 1 Introduction

The discrimination between benign and malignant pulmonary nodules is pivotal for oncological diagnosis and treatment planning [1]. Traditional methodologies frequently hinge on histopathological confirmation or adherence to guidelines such as the Fleischner Society Guidelines, 2017 [2]. Nevertheless, these methods can be both invasive and time consuming. Radiology AI‐enabled biomarkers represent an emerging field that focuses on extracting measurable characteristics from medical images and transforming them into analyzable data [3, 4]. This approach avoids invasive procedures, and the diagnostic performance of AI‐enabled biomarkers has been demonstrated to match or even surpass that of human experts [5].

Research on AI‐enabled biomarkers primarily falls into two categories: hand‐crafted radiomics and deep learning (DL) approaches [6]. In the field of hand‐crafted radiomics, computer scientists, radiologists, and oncologists continuously define new feature representations composed of feature measurements with specific algorithmic derivations. The main focus is on various attributes within the tumor area, such as shape or texture, as well as the tumor microenvironment, including texture and tumor vasculature [7]. In DL approaches, feature representations are automatically learned by neural networks. Currently, there is a considerable number of research focusing on extracting AI‐enabled biomarkers from intranodular and perinodular regions. However, investigation into adipose tissue region is exceedingly scarce [8].

Recent studies have demonstrated that adipose tissue inflammation is associated with cancer incidence and progression [9]. Obesity leads to increased damage to deoxyribonucleic acid (DNA) and reduced DNA repair in both animal models and humans [10, 11]. The inflammatory white adipose tissue provides adipokines and secretes pro‐angiogenic molecules to accelerate tumor metastasis within the microenvironment [12]. Furthermore, recent studies have revealed that adipose tissue releases extracellular vesicles to mediate the communication between tumor cells and their microenvironment [13, 14]. Numerous studies have revealed the association between adipose tissue and cancer [9, 10, 11, 12, 13, 14]. However, research incorporating AI‐enabled biomarkers from adipose tissue radiomics into nomograms and further investigating their supplementary value has yet to be found.

To address this issue, we extracted intrathoracic adipose tissue radiomics features and combined them with intranodular and perinodular region (IPN) deep learning features and clinical characteristics to construct a deep learning radiomics clinical nomogram (DLRCN). While numerous studies have focused on the malignant diagnosis of pulmonary nodules, solely investigating the benign‐malignant nature of pulmonary nodules is one‐sided [15]. Multiple lines of evidence have indicated that subsolid pulmonary nodules represent a fundamentally distinct disease compared to solid pulmonary nodules, exhibiting different etiology, genetic patterns, and clinical behavior [16, 17]. Therefore, this study, in addition to evaluating the performance of DLRCN in determining malignancy of pulmonary nodules, further delineated these nodules into subgroups of solid and subsolid pulmonary nodules for detailed analysis.

## 2 Materials and Methods

### 2.1 Patients

This retrospective study was carried out after approval from the Institutional Review Board of three centers (Harbin Medical University Cancer Hospital, the First Affiliated Hospital of Harbin Medical University, and the Second Affiliated Hospital of Harbin Medical University). Because of the retrospective nature of the research, the requirement for informed consent was waived.

**FIGURE 1:**
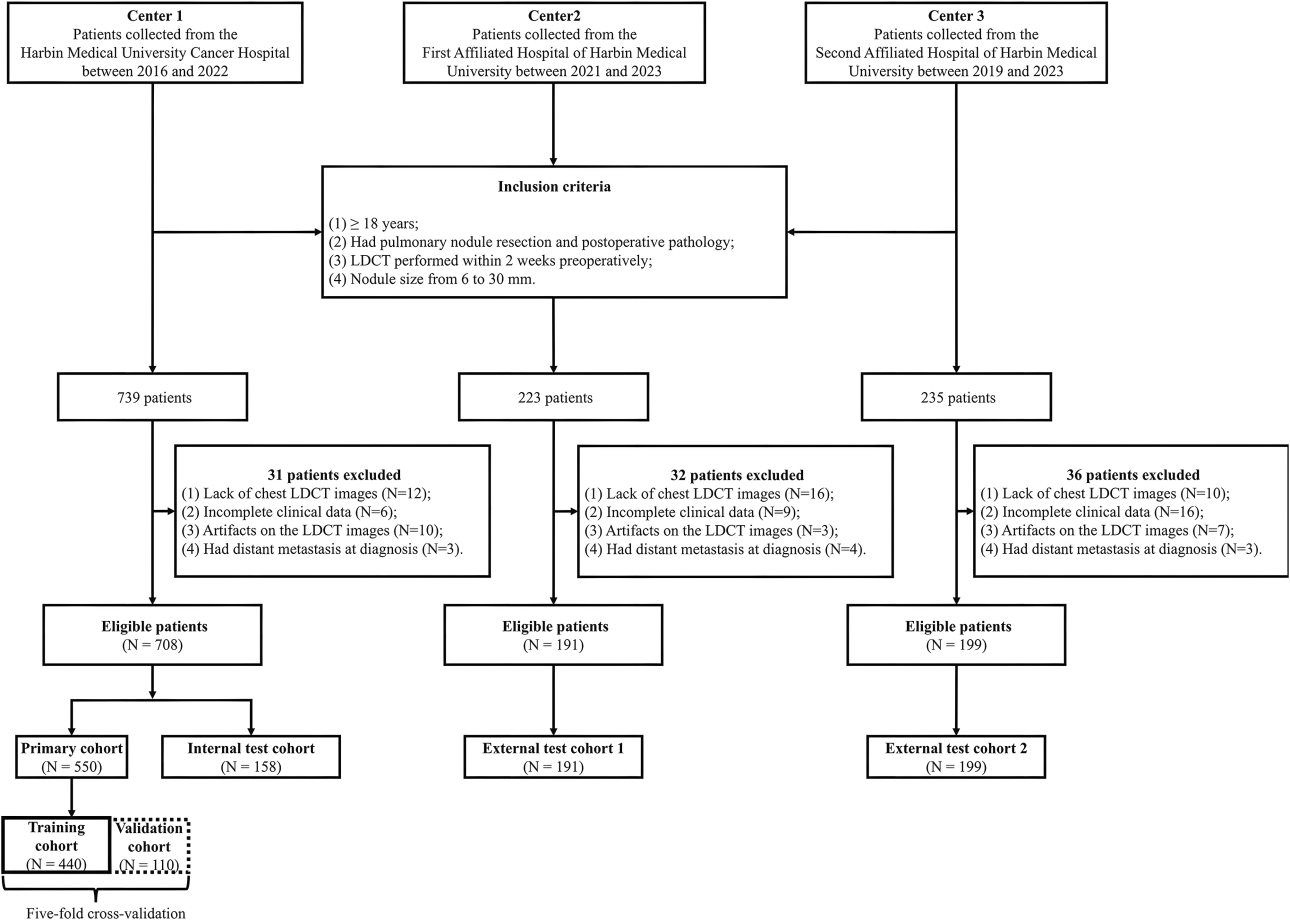
Flowchart for patient inclusion. LDCT, low‐dose computed tomography.

As illustrated in Figure 1 and Supporting Information A1, a total of 1098 patients with pulmonary nodules from three centers were divided into four cohorts: a primary cohort (PC) for training (*N* = 550), an internal test cohort (I‐T, *N* = 158), and two external test cohort (E‐T1, *N* = 191, E‐T2, *N* = 199).

The inclusion criteria for this study were as follows: (a) subjects had to be older than 18 years; (b) all patients underwent pulmonary nodule resection, and postoperative pathology was performed; (c) a low‐dose computed tomography (LDCT) scan was carried out less than 2 weeks before surgery; (d) the LDCT measurement diameter of nodules ranges from 6 to 30 mm. The exclusion criteria are as follows: (I) preoperative treatment; (II) incomplete baseline data; (III) a lack of chest LDCT images or there are artifacts on the LDCT image that affect the evaluation; (IV) distant metastasis at diagnosis.

### 2.2 Data Collection

The following clinical characteristics were collected: age, sex, body mass index (BMI), current/former smoke, hypertension, heart disease, family cancer history, and previous cancer history. The following LDCT features of pulmonary nodules were collected: (a) the location of the pulmonary nodules; (b) the size of the pulmonary nodules; (c) the presence of a spiculated sign (yes or no); (d) the lesion shape (round/oval or irregular); (e) the presence of lobulated shape (yes or no); (f) the demarcation of the pulmonary nodules (clear or fuzzy). Chest LDCT images from three hospitals were collected. The LDCT image acquisition settings of each hospital were presented in Supporting Information A2 and Table S1.

### 2.3 Procedures

**FIGURE 2:**
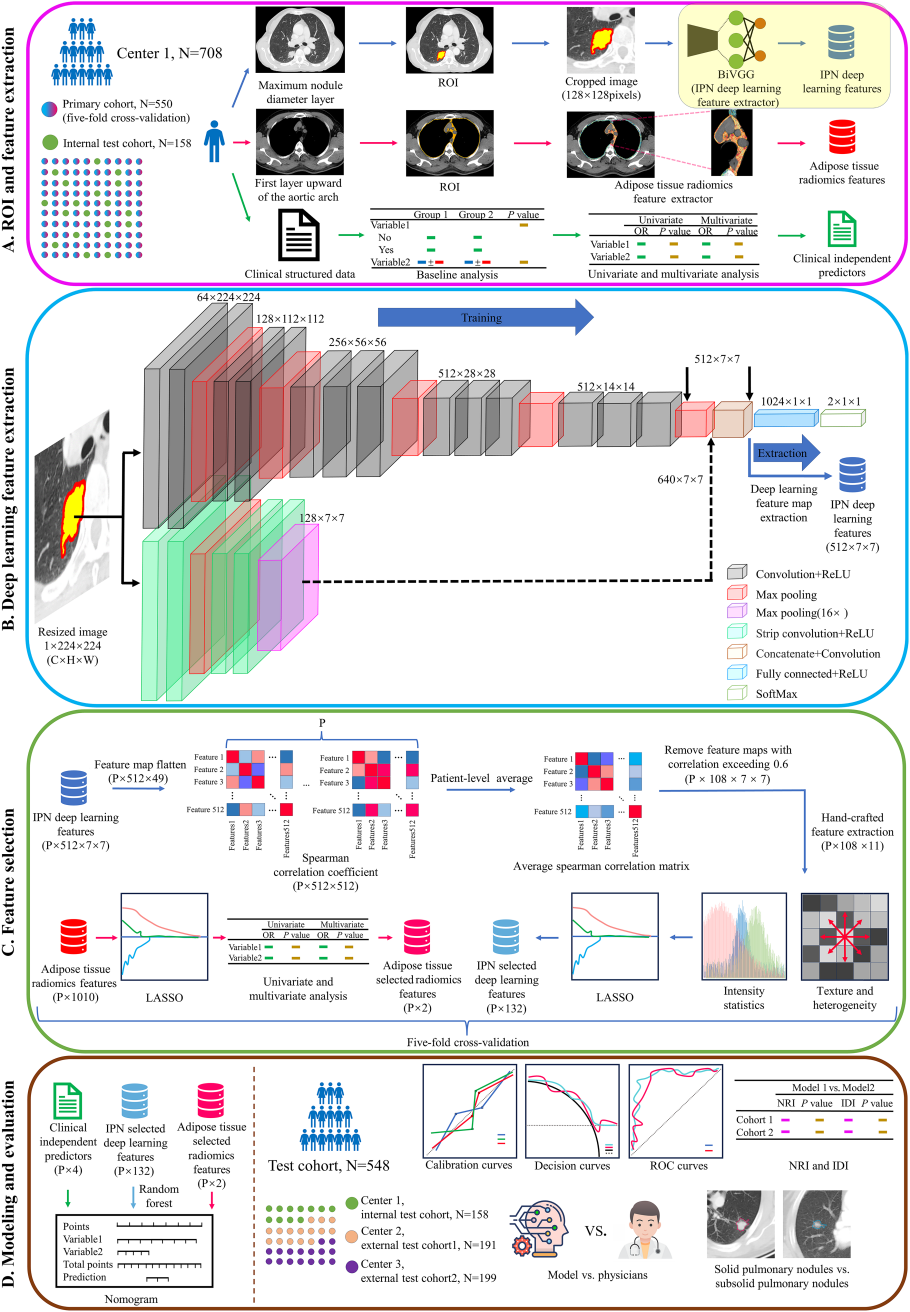
Modeling procedure. ROI, region of interest; OR, odds ratio; ReLU, rectified linear unit; IPN, intranodular and perinodular region; P, patient count; C, channel; H, height; W, width; ROC, receiver operating curve; NRI, net reclassification index; IDI, integrated discrimination improvement.

As illustrated in Figure 2. A total of 708 patients from Center 1 were allocated to the PC and I‐T. The PC was utilized for performing five‐fold cross‐validation to train the model. The maximum nodule diameter layer and the first layer above the aortic arch were selected from the preoperative LDCT scan. Radiologists delineated the corresponding region of interest (ROI) of IPN and adipose tissue. For feature extraction of the IPN, we constructed a deep learning model named bilateral VGG (BiVGG). For the adipose tissue feature extraction, we employed radiomics feature extraction approaches, acquiring features with radiomics nomenclature. Regarding clinical characteristics, to begin with, we performed baseline analysis to identify inter‐group differences. Subsequently, univariate analysis was conducted to determine features with statistically significant associations. Finally, multivariate analysis was employed to identify independent predictors.

Figure 2B provided an enlarged view of the highlighted area in Figure 2A, illustrating the bilateral structure of BiVGG. Notably, during training, BiVGG underwent SoftMax layer processing for loss calculation; however, during feature extraction, feature maps were directly obtained from the output of the final convolutional layer without passing through fully connected layers or SoftMax.

Figure 2C illustrates our feature selection process. First and foremost, Spearman correlation analysis was applied to eliminate redundancy between deep learning feature maps. Additionally, hand‐crafted radiomics feature extraction approaches were employed to obtain deep learning radiomics features, building upon the selected feature maps. Finally, the least absolute shrinkage and selection operator (LASSO) regression was utilized to obtain the selected IPN features. For adipose tissue radiomics features, in addition to employing LASSO regression for feature selection, these features were integrated with clinical characteristics to conduct both univariate and multivariate analyses to identify independent predictors.

As shown in Figure 2D, adipose tissue selected radiomics features and clinical independent predictors were directly used as signatures of nomogram, and the IPN deep learning features underwent a random forest (RF) model to obtain the IPN signature. Constructing DLRCN using the signatures mentioned above. Patients from three different centers constituted the test cohort. We evaluated the DLRCN's calibration, clinical usefulness, receiver operating characteristic (ROC), net reclassification improvement (NRI), and integrated discrimination improvement (IDI). Comparison with radiologists and subgroups analysis were also considered in the evaluation.

### 2.4 Scanning Procedure

LDCT was performed with subjects in the supine position. The location and imaging information of pulmonary nodules on LDCT images were selected and annotated by experienced radiologists. The ROI of the IPN was delineated by a radiologist with 10 years of experience. After 3 months, 60 LDCT images were randomly selected, and another radiologist with 5 years of experience re‐delineated the ROI. The interclass correlation coefficient (ICC) was then calculated (Supporting Information A3).

### 2.5 Region of Interest Segmentation

The first layer upward of the aortic arch was visually identified, and then the first axial image above the arch was selected by scrolling toward the apex of the lungs. With semi‐automatic segmentation, it enabled the inclusion of the maximum possible area. Pixels with Hounsfield unit (HU) values between −200 and − 40 within the selected region were defined as adipose tissue in the thorax. Within the region of interest, Adipose tissue at the first layer upward of the aortic arch was delineated by Image J 1.53A software (Wayne Rasband and contributors, National Institutes of Health, USA), the ICC analysis was conducted as previously described (all ICC > 0.7).

### 2.6 Deep Learning Feature Extraction

The BiVGG was utilized to extract 512 deep learning feature maps from the final convolutional layer of each ROI. After that, we eliminated redundancy between feature maps using Spearman correlation analysis. Finally, we further employed traditional hand‐crafted radiomics feature extraction methods. For each feature map, 11 radiomics features were extracted using specific formulas, as illustrated in Supporting Information A4.

### 2.7 Feature Selection

Feature selection was performed in PC. It is worth noting that our entire experiment employed five‐fold cross‐validation, during the training of the BiVGG model, we trained five models, each corresponding to one‐fold of the experiment. Consequently, the results of feature selection varied for each fold. However, during the feature selection phase, it was necessary to filter a unified and representative set of feature indices. To address this, we employed the “Take it all” method, combining the feature selection subsets from each fold into a comprehensive collection to guide every experiment fold [18]. The specific results of feature selection for each fold, as well as the final outcome were presented in Table S2. The detailed feature selection methods were elucidated in Supporting Information A5.

### 2.8 DLRCN Construction

Signature establishment was also conducted in PC. A comparison of three methods, namely, support vector machine, logistic regression, and RF, was performed, and the most effective one was chosen to construct an IPN signature. Additionally, a grid search was utilized to determine the optimal combination of model hyperparameters. Adipose tissue radiomics features and clinical characteristics that align with the nature of independent predictors were also incorporated into the DLRCN. Logistic regression was employed to integrate the aforementioned signatures.

### 2.9 DLRCN Evaluation

We assessed the performance of DLRCN in the I‐T, E‐T1, and E‐T2. Calibration curves were employed to visualize the Hosmer‐Lemeshow goodness‐of‐fit test across different cohorts, and decision curve analysis (DCA) was used to evaluate the clinical utility of the model. It is noteworthy that in the ROC curve analysis, we incorporated the results of three radiologists with an average of 6 years of experience (ranging from 3 to 10 years) in diagnosing the benign or malignant nature of pulmonary nodules. The specific number of diagnostic errors was also presented. For the supplementary value of adipose tissue radiomics features, we utilized NRI and IDI analysis. Finally, we further explored the supplementary value of adipose tissue radiomics features in subgroup analysis.

### 2.10 Statistical Analysis

Using R software (version 4.1.0; http://www.Rproject.org; R Foundation for Statistical Computing, Vienna, Austria) and SPSS software (version 25; https://www.ibm.com/products/spss‐statistics; IBM, SPSS; Chicago, IL) in statistical analysis. In the univariate analysis, the differences in clinical characteristics between the patients in different groups were assessed using independent *t* test or Manne–Whitney *U* test for continuous variables and Fisher's exact test or chi‐square test for categorical variables. Analysis of variance and Kruskal–Wallis *H* test were implemented to compare the differences between the two groups. The significance of area under the curve (AUC) differences was estimated using 95% CI or Delong test. *p* value less than 0.05 was considered statistically significant. We conducted five‐fold cross‐validation to train and validate the model. Performance indicators included sensitivity, specificity, accuracy, and AUC.

## 3 Results

### 3.1 Baseline Analyses

As shown in Table 1, all four cohorts were divided into subgroups. In the PC, there were a total of 550 patients with pulmonary nodules, including 256 patients with solid nodules [117 benign (45.7%); 139 malignant (54.3%); 150 female (58.6%); 106 male (41.4%); mean ± std. age, 56.1 ± 9.1; mean ± std. nodule size, 18.1 ± 9.6] and 294 patients with subsolid nodules [113 benign (38.4%); 181 malignant (61.6%); 172 female (58.5%); 122 male (41.5%); mean ± std. age, 55.7 ± 9.5; mean ± std. nodule size, 17.0 ± 7.6]. There were no significant differences between the subsolid and solid groups (*p* > 0.05). Additionally, the I‐T, E‐T1, and E‐T2 were generally consistent with the PC.

**TABLE 1:** Baseline data.

Variables	Primary cohort (*N* = 550)			Internal test cohort (*N* = 158)			External test cohort 1 (*N* = 191)			External test cohort 2 (*N* = 199)		
	Solid^a^ (*N* = 256)	Sub solid^a^ (*N* = 294)	*p*	Solid^a^ (*N* = 87)	Sub solid^a^ (*N* = 71)	*p*	Solid^a^ (*N* = 82)	Sub solid^a^ (*N* = 109)	*p*	Solid^a^ (*N* = 88)	Sub solid^a^ (*N* = 111)	*p*
Histology			0.102			1.000			0.633			0.629
Benign	117 (45.7)	113 (38.4)		15 (17.2)	13 (18.3)		19 (23.2)	21 (19.3)		20 (22.7)	21 (18.9)	
Malignant	139 (54.3)	181 (61.6)		72 (82.8)	58 (81.7)		63 (76.8)	88 (80.7)		68 (77.3)	90 (81.1)	
Sex			1.000			0.682			0.012			0.148
Female	150 (58.6)	172 (58.5)		50 (57.5)	44 (62.0)		42 (51.2)	35 (32.1)		44 (50.0)	68 (61.3)	
Male	106 (41.4)	122 (41.5)		37 (42.5)	27 (38.0)		40 (48.8)	74 (67.9)		44 (50.0)	43 (38.7)	
BMI (kg/m^2^)	24.3 ± 3.8	24.5 ± 3.2	0.481	24.8 ± 3.0	24.4 ± 2.7	0.361	24.9 ± 3.3	22.7 ± 4.6	< 0.001	24.2 ± 3.3	23.3 ± 2.9	0.075
Age (years)	56.1 ± 9.1	55.7 ± 9.5	0.626	58.6 ± 8.5	56.8 ± 9.5	0.221	56.4 ± 10.2	57.6 ± 9.9	0.447	59.8 ± 8.4	56.9 ± 9.6	0.023
Smoke			0.054			0.559			< 0.001			0.014
Never	171 (66.8)	216 (73.5)		62 (71.3)	55 (77.4)		38 (46.3)	108 (99.1)		65 (73.9)	94 (84.7)	
Former	30 (11.7)	18 (6.1)		10 (11.5)	8 (11.3)		2 (2.5)	0 (0.0)		11 (12.5)	14 (12.6)	
Now	55 (21.5)	60 (20.4)		15 (17.2)	8 (11.3)		42 (51.2)	1 (0.9)		12 (13.6)	3 (2.7)	
Hypertension			0.949			0.099			0.675			0.729
No	204 (79.7)	236 (80.3)		72 (82.8)	50 (70.4)		69 (84.1)	88 (80.7)		73 (83.0)	88 (79.3)	
Yes	52 (20.3)	58 (19.7)		15 (17.2)	21 (29.6)		13 (15.9)	21 (19.3)		15 (17.0)	22 (20.7)	
Heart disease			0.861			0.755			1.000			0.517
No	243 (94.9)	277 (94.2)		80 (92.0)	67 (94.4)		75 (91.5)	100 (91.7)		85 (96.6)	104 (93.7)	
Yes	13 (5.1)	17 (5.8)		7 (8.0)	4 (5.6)		7 (8.5)	9 (8.3)		3 (3.4)	7 (6.3)	
Family cancer history			0.552			0.815			0.357			0.085
No	223 (87.1)	262 (89.1)		75 (86.2)	63 (88.7)		79 (96.3)	101 (92.7)		85 (96.6)	111 (100.0)	
Yes	33 (12.9)	32 (10.9)		12 (13.8)	8 (11.3)		3 (3.7)	8 (7.3)		3 (3.4)	0 (0.0)	
Cancer history			0.048			0.691			0.010			0.695
No	243 (94.9)	289 (98.3)		83 (95.4)	69 (97.2)		73 (89.0)	107 (98.2)		86 (97.7)	107 (96.4)	
Yes	13 (5.1)	5 (1.7)		4 (4.6)	2 (2.8)		9 (11.0)	2 (1.8)		2 (2.3)	4 (3.6)	
Nodule size (mm)	18.1 ± 9.6	17.0 ± 7.6	0.132	17.8 ± 6.9	14.3 ± 4.8	< 0.001	16.7 ± 6.8	11.6 ± 3.2	0.003	18.1 ± 6.7	12.8 ± 4.9	< 0.001
Location			0.089			0.009			< 0.001			0.454
Right												
Upper lobe	77 (30.1)	111 (37.8)		23 (26.4)	29 (40.8)		15 (18.3)	0 (0.0)		26 (29.5)	43 (38.7)	
Middle lobe	29 (11.2)	24 (8.2)		9 (10.3)	5 (7.1)		4 (4.9)	7 (6.4)		12 (13.6)	9 (8.1)	
Lower lobe	48 (18.8)	50 (17.0)		17 (19.5)	3 (4.2)		0 (0.0)	3 (2.8)		8 (9.1)	8 (7.2)	
Left												
Upper lobe	55 (21.5)	73 (24.8)		19 (21.9)	24 (33.8)		60 (73.1)	97 (89.0)		22 (25.1)	22 (19.8)	
Lower lobe	47 (18.4)	36 (12.2)		19 (21.9)	10 (14.1)		3 (3.7)	2 (1.8)		20 (22.7)	29 (26.2)	
Spiculated sign			0.155			0.024			< 0.001			0.022
No	230 (89.8)	275 (93.5)		78 (89.7)	70 (98.6)		62 (75.6)	109 (100.0)		69 (78.4)	101 (91.0)	
Yes	26 (10.2)	19 (6.5)		9 (10.3)	1 (1.4)		20 (24.4)	0 (0.0)		19 (21.6)	10 (9.0)	
Lesion shape			0.007			0.011			0.227			0.619
Round/oval	106 (41.4)	88 (29.9)		27 (31.0)	9 (12.7)		3 (3.7)	10 (9.2)		24 (27.3)	35 (31.5)	
Irregular	150 (58.6)	206 (70.1)		60 (69.0)	62 (87.3)		79 (96.3)	99 (90.8)		64 (72.7)	76 (68.5)	
Lobulated shape			0.001			0.135			0.014			0.016
No	210 (82.0)	269 (91.5)		73 (83.9)	66 (93.0)		77 (93.9)	109 (100.0)		77 (87.5)	108 (97.3)	
Yes	46 (18.0)	25 (8.5)		14 (16.1)	5 (7.0)		5 (6.1)	0 (0.0)		11 (12.5)	3 (2.7)	
Demarcation			0.200			0.973			0.843			0.767
Clear	77 (30.1)	73 (24.8)		16 (18.4)	12 (16.9)		13 (15.9)	15 (13.8)		32 (36.4)	37 (33.3)	
Fuzzy	179 (69.9)	221 (75.2)		71 (81.6)	59 (83.1)		69 (84.1)	94 (86.2)		56 (63.6)	74 (66.7)	

Abbreviation: BMI, body mass index.

^a^ Data in parentheses are percentage.

### 3.2 Feature Extraction and Selection

During the deep learning feature extraction phase, the VGG16 model was selected as the base model. Building upon this foundation, we refined the architecture of the VGG16 model and labeled it as BiVGG. As shown in Table S3, the performance and generalization of the BiVGG model were generally superior to other base models. After confirming BiVGG as the deep learning feature extraction model for IPN. We extracted 512 deep learning feature maps from the last convolutional layer of the BiVGG network.

After that, we encountered the challenge of redundancy in the extracted feature maps. To address this issue, we calculated Spearman correlation coefficients between different feature maps and determined a correlation threshold of 0.6 to be the standard for eliminating redundancy. Following this step, we identified 108 deep learning feature maps and computed 11 radiomics features for each feature map. The corresponding radiomics formulas are provided in the Supporting Information A4. Finally, LASSO regression determined 132 selected IPN deep learning features, as illustrated in Figure S1.

For adipose tissue radiomics feature extraction, we initially extracted 1010 radiomics features and conducted preliminary screening using LASSO regression (Figure S2), resulting in 10 selected adipose tissue radiomics features. Subsequently, as shown in Table 2, we combined clinical features with adipose tissue radiomics features for univariate logistic regression analysis. In this step, we used a significance level of *p* < 0.05 as the inclusion criterion. Among the clinical features, sex, age, lesion shape, and demarcation exhibited significant differences, while 10 selected adipose tissue radiomics features also demonstrated significant differences. After that, we conducted a multivariate analysis on the above features. All clinical features showed characteristics of independent predictors (*p* < 0.05). However, eight selected adipose tissue radiomics features lack of independence (*p* > 0.05), ultimately leaving two selected adipose tissue radiomics features with the nature of independent predictors which were exponential_glszm_SizeZoneNonUniformityNormalized and gradient_glrlm_RunLengthNonUniformityNormalized.

**TABLE 2:** Univariate and multivariate analysis.

Variables	Univariate		Multivariate	
	OR^a^	*p*	OR^a^	*p*
Sex (Male vs. Female)	0.453 (0.319,0.640)	0.001	0.299 (0.118,0.720)	0.009
BMI (kg/m^2^)	1.014 (0.966,1.066)	0.571		
Age (years)	1.034 (1.015,1.054)	0.001	1.070 (1.020,1.127)	0.007
Smoke				
Former versus Never	0.850 (0.465,1.571)	0.598		
Now versus Never	0.721 (0.474,1.097)	0.126		
Hypertension (Yes vs. No)	0.911 (0.598,1.393)	0.666		
Heart disease (Yes vs. No)	1.257 (0.595,2.781)	0.557		
Family cancer history (Yes vs. No)	1.089 (0.646,1.864)	0.752		
Cancer history (Yes vs. No)	0.895 (0.348,2.379)	0.818		
Nodule size (mm)	0.955 (0.934,0.976)	0.001	0.975 (0.916,1.030)	0.394
Location				
Left lower lobe versus Left upper lobe	0.409 (0.231,0.717)	0.002	0.931 (0.221,3.816)	0.921
Right upper lobe versus Left upper lobe	1.087 (0.677,1.738)	0.729	1.208 (0.392,3.736)	0.741
Right middle lobe versus Left upper lobe	1.091 (0.561,2.168)	0.800	0.658 (0.131,3.509)	0.615
Right lower lobe versus Left upper lobe	0.457 (0.266,0.780)	0.004	0.547 (0.145,1.986)	0.362
Spiculated sign (Yes vs. No)	1.085 (0.587,2.052)	0.796		
Lesion shape (Irregular vs. Round/oval)	9.849 (6.599,14.928)	0.001	3.264 (1.282, 8.730)	0.015
Lobulated shape (Yes vs. No)	0.662 (0.401,1.092)	0.105		
Demarcation (Fuzzy vs. Clear)	11.139 (7.113,17.934)	0.001	12.418 (3.399,57.162)	0.001
original_glszm_SizeZoneNonUniformity	0.977 (0.974,0.0.980)	0.001	1.008 (0.996, 1.021)	0.190
exponential_glszm_GrayLevelNonUniformityNormalized	2.021 (1.323,4.876)	0.001	2.066 (0.614,7.929)	0.091
exponential_glszm_SizeZoneNonUniformityNormalized	0.194 (0.082,0.311)	0.001	0.194 (0.176,0.233)	0.016
gradient_glrlm_RunLengthNonUniformityNormalized	0.807 (0.155,0.961)	0.001	0.233 (0.161,0.448)	0.028
wavelet.LH_glszm_SmallAreaEmphasis	0.696 (0.628,0.763)	0.001	1.029 (0.326,9.447)	0.673
wavelet.HL_firstorder_Minimum	0.897 (0.879,0.912)	0.001	0.964 (0.928,0.999)	0.069
wavelet.HH_firstorder_Mean	0.298 (0.282,0.402)	0.001	1.961 (0.922,2.008)	0.092
wavelet.HH_glszm_ZoneEntropy	0.589 (0.379,0.630)	0.001	0.402 (0.049,1.315)	0.225
wavelet.HH_ngtdm_Busyness	0.355 (0.300,0.413)	0.001	0.786 (0.535,1.136)	0.207
wavelet.LL_glrlm_RunEntropy	1.479 (1.257,1.698)	0.001	1.253 (0.366,1.870)	0.283

Abbreviations: BMI, body mass index; OR, odds ratio.

^a^ Data in brackets are 95% confidence intervals.

Finally, we identified 4 clinical independent predictors, 2 adipose tissue radiomics features were qualified as independent predictors, and 132 IPN deep learning features.

### 3.3 Deep Learning Signature Building

RF was identified as the optimal choice to construct IPN signature (Table S4). Additionally, based on grid search, we determined that the hyperparameter combination of n‐estimators = 25, min‐samples‐leaf = 13, and max‐depth = 6 could harness the best performance of RF (Table S5).

### 3.4 DLRCN Construction and Evaluation

**FIGURE 3:**
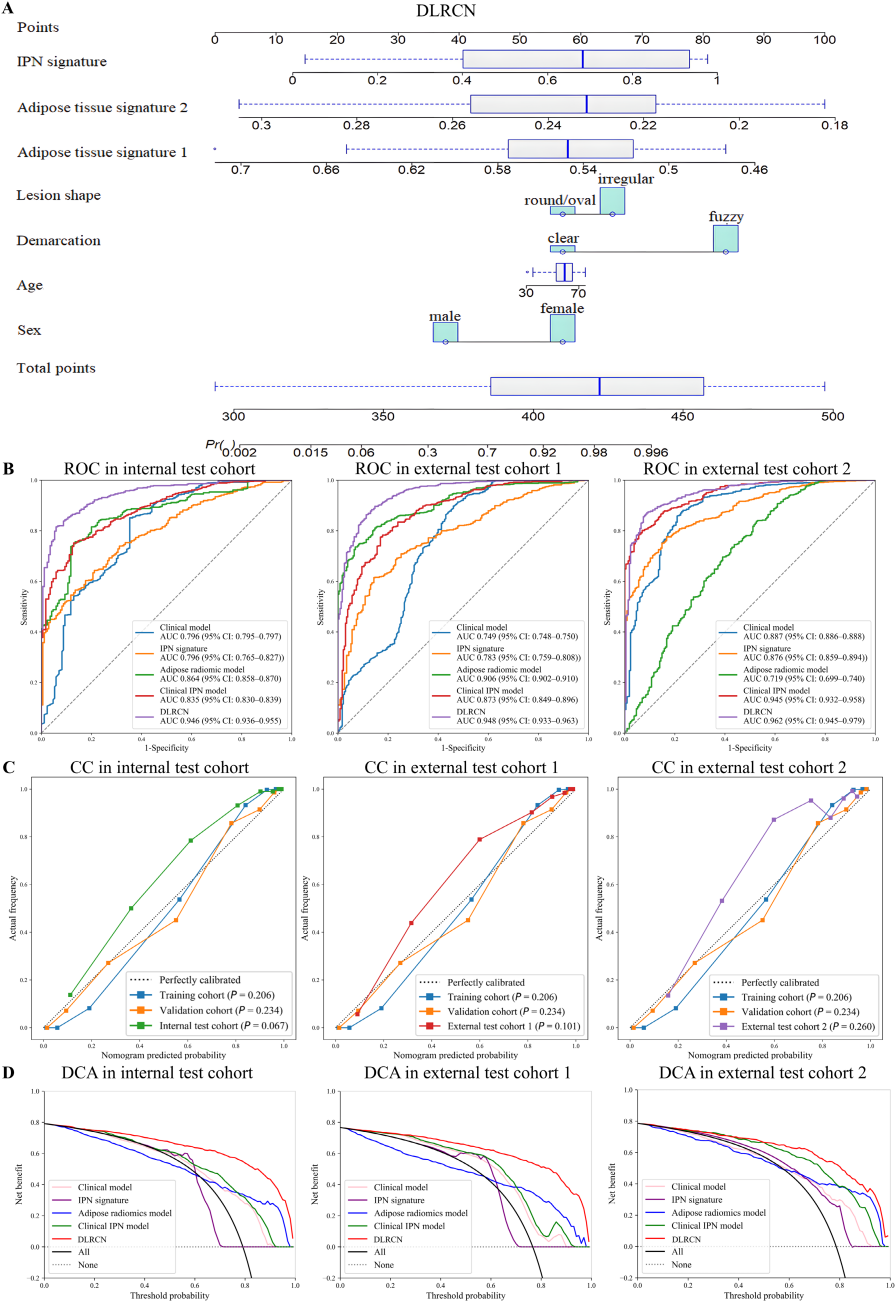
DLRCN and its performance. (A) Deep learning radiomics clinical nomogram (DLRCN) with intranodular and perinodular region (IPN) signature, adipose tissue signatures, and clinical signatures; (B) receiver operating curve (ROC) of DLRCN in all cohorts; (C) calibration curves (CC) of DLRCN in three test cohorts; (D) decision curve analysis (DCA) for DLRCN, clinical IPN model, Adipose radiomics model, IPN signature, clinical model, “All,” “None” scheme in three test cohorts.

Clinical independent predictors were directly used as signatures, adipose tissue independent predictors were renamed as adipose tissue signature 1 and adipose tissue signature 2, and IPN signatures were integrated into the DLRCN by logistic regression (Figure 3A). The coefficients of logistic regression were reflected in Table S6. As shown in Table 3, in I‐T, the AUC of DLRCN reached 0.946, which was significantly higher than the AUC of clinical model (AUC:0.796, *p* < 0.05, Delong, etc.), IPN signature (AUC: 0.796, *p* < 0.05, Delong, etc.), and adipose radiomics model (AUC: 0.864, *p* < 0.05, Delong, etc.), even compared to the mixed model which combined IPN signature and clinical signatures (AUC:0.835, *p* < 0.05, Delong, etc.). It means that the addition of adipose tissue signatures significantly improved the performance of DLRCN. In E‐T1, the AUC of DLRCN reached 0.948, in E‐T2, DLRCN's AUC achieved the supreme 0.962, and was significantly higher than other models (all *p* < 0.05, Delong, etc.). The corresponding ROC curves are depicted in Figure 3, B. The stratification analysis showed that the performance of our DLRCN was not affected by age, sex, body mass index, version of CT system, and slice thickness (Figures S3 and Supporting Information A6). Calibration curves of the three cohorts revealed remarkable consistency between the DLRCN predictions and the actual benign or malignant status of the nodules, as reflected in the *p* values (Figure 3C).

**TABLE 3:** The predictive performance of single or combined predictors in different cohorts.

	AUC^a^	ACC^a^	SEN^a^	SPE^a^	*p*
Internal test cohort					
Clinical model	0.796 (0.795,0.797)	0.784 (0.749,0.819)	0.828 (0.768,0.888)	0.618 (0.550,0.685)	0.001
IPN signature	0.796 (0.765,0.827)	0.731 (0.700,0.763)	0.746 (0.705,0.787)	0.676 (0.561,0.792)	0.001
Adipose radiomics model	0.864 (0.858,0.870)	0.828 (0.819,0.837)	0.904 (0.878,0.929)	0.541 (0.444,0.638)	0.011
Clinical IPN model	0.835 (0.830,0.839)	0.760 (0.714,0.805)	0.767 (0.693,0.842)	0.729 (0.664,0.795)	0.003
Mayo model	0.561 (0.472,0.642)	0.505 (0.428,0.587)	0.438 (0.398,0.454)	0.685 (0.581,0.937)	0.001
Brock model	0.594 (0.510,0.674)	0.480 (0.473,0.625)	0.449 (0.399,0.657)	0.833 (0.679,0.992)	0.001
DLRCN	0.946 (0.936,0.955)	0.859 (0.837,0.881)	0.853 (0.809,0.897)	0.882 (0.818,0.947)	Reference
External test cohort 1					
Clinical model	0.749 (0.748,0.750)	0.822 (0.810,0.835)	0.923 (0.890,0.957)	0.491 (0.427,0.556)	0.001
IPN signature	0.783 (0.759,0.808)	0.710 (0.679,0.740)	0.698 (0.667,0.729)	0.748 (0.692,0.804)	0.001
Adipose radiomics model	0.906 (0.902,0.910)	0.840 (0.829,0.850)	0.922 (0.901,0.943)	0.570 (0.545,0.594)	0.049
Clinical IPN model	0.873 (0.849,0.896)	0.769 (0.729,0.808)	0.755 (0.689,0.820)	0.813 (0.749,0.877)	0.001
Mayo model	0.580 (0.469,0.684)	0.450 (0.362,0.578)	0.427 (0.304,0.464)	0.818 (0.587,0.891)	0.001
Brock model	0.595 (0.502,0.684)	0.675 (0.446,0.681)	0.736 (0.584,0.863)	0.405 (0.309,0.583)	0.001
DLRCN	0.948 (0.933,0.963)	0.871 (0.860,0.882)	0.875 (0.844,0.907)	0.857 (0.796,0.917)	Reference
External test cohort 2					
Clinical model	0.887 (0.886,0.888)	0.748 (0.692,0.804)	0.723 (0.640,0.805)	0.842 (0.799,0.885)	0.020
IPN signature	0.876 (0.859,0.894)	0.742 (0.715,0.769)	0.703 (0.659,0.746)	0.888 (0.834,0.943)	0.001
Adipose radiomics model	0.719 (0.699,0.740)	0.718 (0.707,0.729)	0.733 (0.701,0.765)	0.765 (0.737,0.794)	0.001
Clinical IPN model	0.945 (0.932,0.958)	0.824 (0.794,0.852)	0.795 (0.759,0.831)	0.930 (0.858,0.999)	0.009
Mayo model	0.562 (0.478,0.643)	0.520 (0.486,0.551)	0.445 (0.329,0.541)	0.677 (0.571,0.773)	0.001
Brock model	0.561 (0.472,0.644)	0.550 (0.436,0.591)	0.467 (0.396,0.579)	0.738 (0.664,0.766)	0.001
DLRCN	0.962 (0.945,0.979)	0.879 (0.853,0.904)	0.871 (0.805,0.937)	0.907 (0.759,0.999)	Reference

Abbreviations: ACC, accuracy; AUC, area under the curve; DLRCN, deep learning radiomics clinical nomogram; IPN, intranodular and perinodular region; SEN, sensitivity; SPE, specificity.

^a^ Data in brackets are 95% confidence intervals.

### 3.5 Clinical Use

If we used this DLRCN to guide pulmonary nodule resection surgeries, as illustrated in Figure 3D, for a wide range of threshold probabilities from 20% to 80%, the DCA indicated that DLRCN could provide greater benefits to patients compared to single signatures, “none” or “all” scheme.

### 3.6 Role of Adipose Tissue

**FIGURE 4:**
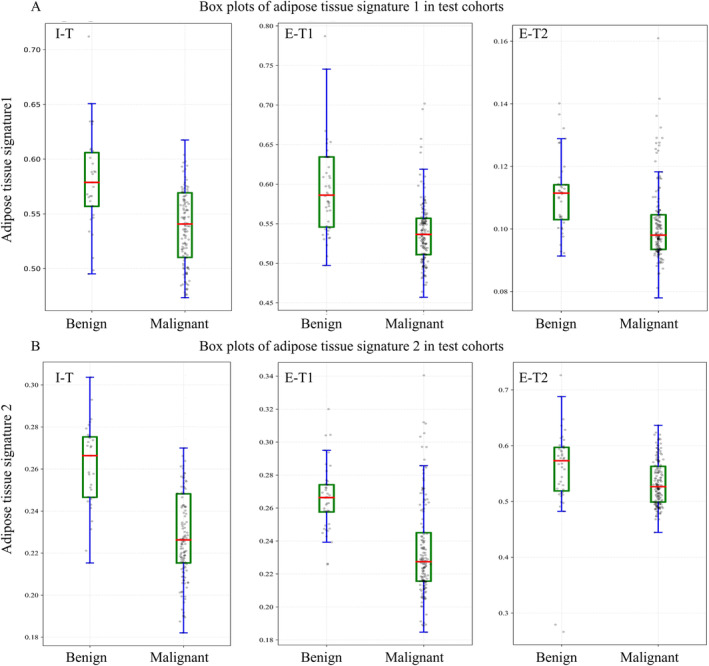
Analysis of the adipose tissue signatures. (A, B) Box plots showing patterns of correlation between malignancy of pulmonary nodules and adipose tissue signatures in internal test cohort (I‐T), external test cohort 1 (E‐T1), and external test cohort 2 (E‐T2).

After the incorporation of adipose tissue signatures, the enhanced performance of DLRCN is evident through the net reclassification improvement (I‐T: NRI = 1.028; E‐T1: NRI = 0.633; E‐T2: NRI = 0.320, all *p* < 0.05) and integrated discrimination improvement (I‐T: IDI = 0.137; E‐T1: IDI = 0.114; E‐T2: IDI = 0.088, all *p* < 0.05). Detailed statistical results are provided in Table S7. As shown in Figure 4, adipose tissue signatures were negatively correlated with the malignancy of pulmonary nodules.

### 3.7 Comparison to Radiologists

**FIGURE 5:**
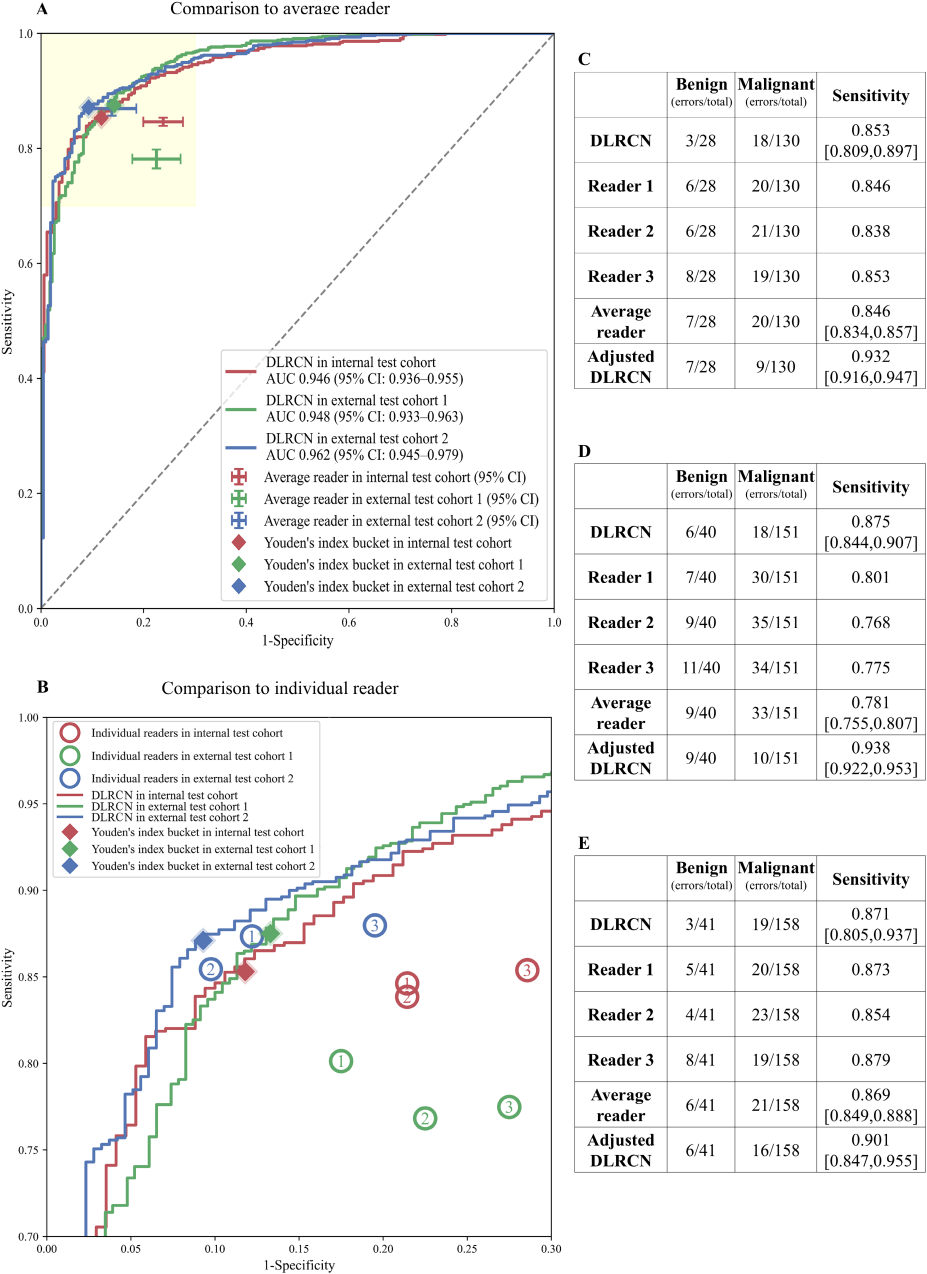
Result of the comparison to radiologists. (A) Performance of deep learning radiomics clinical nomogram (DLRCN) versus average reader in three test cohorts; (B) performance of DLRCN versus individual readers in three test cohorts; (C, D, E) sensitivity and error rate comparison between DLRCN and readers in three test cohort.

We conducted a retrospective reader study with three radiologists (average of 6 years clinical experience, range 3–10 years). Readers were given access to associated patient demographics and clinical history, but without additional information on adipose tissue, while the deep learning model have access to all information. As shown in Figure 5, in I‐T. The ROC of the DLRCN was represented by the red line, with an AUC of 0.946 (95% CI: 0.936, 0.955). The average performance of the three radiologists was lower than DLRCN (Figure 5A). In the magnified image of the yellow area, it can be observed more clearly (Figure 5B). The specific error rates of DLRCN are reflected in Figure 5C. In the benign subgroup, there were a total of 28 cases with 3 prediction errors, while in the malignant subgroup, there were a total of 130 cases with 18 prediction errors, fewer than the average reader error rate (benign 7/28, malignant 20/130). In addition, we used a more intuitive evaluation approach. Initially, we aligned the specificity of DLRCN with the average reader. Subsequently, we compared the sensitivity of adjusted DLRCN with that of the average reader yielded a statistically significant sensitivity boost of 8.6% (95% CI: 8.2%, 9.0%). Similarly, the performance of all three radiologists in the two external test cohorts tended at or below the DLRCN (Figure 5D,E).

### 3.8 Subgroups Analyses

For determining whether adipose tissue radiomics features exhibit divergent supplementary value for predicting malignant pulmonary nodules in subgroups, we conducted a further analysis of the error rates of DLRCN and readers in the two subgroups (Table 4). In the solid subgroup of I‐T, DLRCN exhibited a specificity of 0.957 (95% CI: 0.917, 0.997), outperforming the average reader's 0.933 (95% CI: 0.853, 0.968). Although its sensitivity was lower at 0.817 (95% CI: 0.773, 0.860), it was comparable to the average reader [0.783 (95% CI: 0.751, 0.815)]. In the subsolid subgroup, the model demonstrated a specificity of 0.813 (95% CI: 0.756, 0.871), significantly higher than the average reader's 0.589 (95% CI: 0.413, 0.766), while maintaining a sensitivity of 0.962 (95% CI: 0.943, 0.981), also higher than that of the average reader [0.925 (95% CI: 0.894, 0.955)]. Similarly, in the two external test cohorts, DLRCN demonstrated sensitivity and specificity levels that approximated or surpassed the average reader performance. Afterward, we conducted a detailed analysis of NRI and IDI in both subgroups. Notably, the incorporation of adipose tissue signatures into DLRCN demonstrated a significant and positive enhancement in NRI and IDI, outperforming the clinical IPN model without adipose tissue signatures [solid group: (I‐T: NRI = 1.478; IDI = 0.234, E‐T1: NRI = 0.408; IDI = 0.044, E‐T2: NRI = 0.647; IDI = 0.113, all *p* < 0.05), subsolid group: (I‐T: NRI = 0.867; IDI = 0.121, E‐T1: NRI = 1.024; IDI = 0.165, E‐T2: NRI = 0.091; IDI = 0.012, all *p* < 0.05)]. This improvement was consistently observed across all three test cohorts. Comprehensive results were provided in Table S8.

**TABLE 4:** Subgroup analysis of solid and subsolid pulmonary nodules.

Cohorts	Solid				Subsolid			
	Benign^a^	Malignant^a^	SPE^b^	SEN^b^	Benign^a^	Malignant^a^	SPE^b^	SEN^b^
Internal test cohort								
DLRCN	14/15	58/72	0.957 (0.917,0.997)	0.817 (0.773,0.860)	11/13	54/58	0.813 (0.756,0.871)	0.962 (0.943,0.981)
Reader 1	13/15	55/72	0.866	0.764	9/13	55/58	0.692	0.948
Reader 2	14/15	56/72	0.933	0.778	8/13	53/58	0.615	0.913
Reader 3	14/15	58/72	0.933	0.806	6/13	53/58	0.461	0.913
Average reader	14/15	56/72	0.933 (0.853,0.968)	0.783 (0.751,0.815)	8/13	54/58	0.589 (0.413,0.766)	0.925 (0.894,0.955)
External test cohort 1								
DLRCN	17/19	56/63	0.905 (0.827,0.982)	0.908 (0.874,0.941)	17/21	77/88	0.881 (0.858,0.989)	0.895 (0.803,0.870)
Reader 1	16/19	48/63	0.842	0.762	17/21	73/88	0.809	0.830
Reader 2	15/19	44/63	0.789	0.698	16/21	72/88	0.762	0.818
Reader 3	12/19	48/63	0.632	0.762	17/21	69/88	0.809	0.784
Average reader	14/19	47/63	0.754 (0.591,0.918)	0.741 (0.685,0.796)	17/21	71/88	0.793 (0.753,0.834)	0.811 (0.775,0.846)
External test cohort 2								
DLRCN	19/20	53/68	0.864 (0.734,0.994)	0.794 (0.697,0.892)	19/21	86/90	0.855 (0.772,0.938)	0.962 (0.935,0.990)
Reader 1	17/20	51/68	0.850	0.750	19/21	87/90	0.905	0.967
Reader 2	18/20	49/68	0.900	0.721	19/21	86/90	0.905	0.956
Reader 3	15/20	52/68	0.750	0.765	18/21	87/90	0.857	0.967
Average reader	17/20	51/68	0.833 (0.719,0.948)	0.745 (0.712,0.779)	19/21	87/90	0.889 (0.847,0.931)	0.963 (0.954,0.973)

Abbreviations: DLRCN, deep learning radiomics clinical nomogram; SEN, sensitivity; SPE, specificity.

^a^ Data represent correct/total.

^b^ Data in brackets are 95% confidence intervals.

## 4 Disscussion

Evidence from both the American National Lung Cancer Screening Trial (NLST) and the European NELSON trial has shown that screening high‐risk individuals with LDCT effectively reduces lung cancer mortality [19, 20]. The existing LDCT‐based protocol for discriminating between benign and malignant nodules primarily relies on factors such as nodule size, density, morphology, and growth rate over time [21]. Previous studies suggest a strong association between nodule size and the probability of lung cancer. The risk of malignancy is less than 1% for nodules < 6 mm, even in high‐risk patients. For nodules between 6 and 20 mm, the risk of malignancy can vary from 8% to 64% [22]. Consequently, the Fleischner Society recommends a cut‐off diameter of 6 mm on LDCT imaging for routine follow‐up [2]. However, risk assessment and management of pulmonary nodules larger than 6 mm have not been well‐established [23]. Current management typically involves watchful waiting with frequent CT follow‐ups. Based on the above research, the risk assessment and subsequent clinical management of the numerous 6–30 mm pulmonary nodules detected by LDCT remain highly challenging. A crucial objective is to enhance the classification accuracy of the existing LDCT‐based protocol by integrating dependable biomarkers.

Through the comparison of lung cancer patients and healthy controls, it has been established that the development of lung cancer is associated with certain adipose factors, such as crosstalk between adipose tissue and cancer cells leads to changes in adipocyte function and paracrine signaling, promoting a microenvironment that supports tumor growth [24, 25]. In this study, we focused on the utility of adipose tissue radiomics for discriminating between benign and malignant. The DLRCN, incorporating adipose tissue signatures in this investigation, demonstrated superior performance in malignancy prediction, achieving AUCs exceeding 0.90 across three test cohorts. Furthermore, the heightened discriminative capability of the DLRCN was validated through improved NRI and IDI, affirming that this enhancement stemmed from feature integration rather than model overfitting. These results collectively emphasize the potential of adipose tissue to provide invaluable insights into the tumor microenvironment, establishing it as a supplementary tool for the prediction of malignant nodules.

Numerous studies have investigated computer‐aided diagnosis (CAD) algorithms for distinguishing between benign and malignant pulmonary nodules. Hempel et al. [26] evaluated the interobserver agreement between deep learning models and radiologists and achieved a kappa value of 0.84. Shen et al. [27] introduced a three‐dimensional deep learning model and compared its performance with that of a radiologist, achieving an AUC of 0.913 (95% CI: 0.885–0.940), sensitivity of 86.1%, and specificity of 83.8% at the optimal decision point. However, prior research has required radiologist involvement in the CAD process, and independent external tests were scarce. In contrast, our proposed DLRCN presented performs human‐independent classification and rigorously evaluated its performance across external test cohorts from two centers. It achieved the highest AUC of 0.962 in a completely independent E‐T2, with error rates significantly lower than those of the average reader (benign: 3/41 vs. 6/41, malignant: 19/158 vs. 21/158). We introduced another comparative method that more intuitively reflects this conclusion [Sensitivity: 0.901 (95% CI: 0.847, 0.955) vs. 0.869 (95% CI: 0.849, 0.888)].

In practical clinical experiments, it is worth noting that, due to the higher risk of malignancy associated with subsolid nodules compared to solid nodules [28]. It is imperative to segregate solid and subsolid nodules for discussion. This issue was meticulously addressed in our experiment to ascertain whether adipose tissue radiomics features exhibit divergent supplementary value for predicting malignant pulmonary nodules in subgroup analyses. The NRI and IDI for the subgroups compellingly confirmed that our adipose tissue radiomics features hold significant supplementary value of malignancy for both solid and subsolid pulmonary nodules.

Several limitations must be acknowledged in this study. Firstly, the delineation of the region of interest was conducted in a single slice (2D), which might not comprehensively represent the entire nodule or adipose tissue. Therefore, there is a necessity for 3D analysis. Secondly, given the retrospective nature of this study, a prospective clinical study is required to validate the generalizability of our findings. Finally, additional research is warranted to explore the potential mechanisms underlying the predictive role of fat features.

## 5 Conclusion

In summary, we integrated deep learning and radiomics to extract various AI‐enabled biomarkers as signatures for the DLRCN. This DLRCN has the potential to become a powerful tool for managing pulmonary nodules. Furthermore, the DLRCN that incorporates adipose tissue signatures demonstrated superior performance compared to the IPN signature alone. Our study sheds light on the key role of adipose tissue. Further studies are warranted.

## Author Contributions

**Shidi Miao:** conceptualization (lead), funding acquisition (lead), investigation (lead), project administration (lead), resources (lead), supervision (lead), writing – review and editing (lead). **Qifan Xuan:** formal analysis (lead), investigation (lead), methodology (lead), validation (lead), visualization (lead), writing – original draft (lead), writing – review and editing (lead). **Hanbing Xie:** data curation (equal), validation (equal), visualization (equal), writing – original draft (equal). **Yuyang Jiang:** data curation (equal), validation (equal), visualization (equal), writing – original draft (equal). **Mengzhuo Sun:** methodology (equal), validation (equal), visualization (equal), writing – original draft (equal). **Wenjuan Huang:** investigation (equal), supervision (equal), writing – review and editing (equal). **Jing Li:** data curation (equal), formal analysis (equal), investigation (equal), supervision (equal). **Hongzhuo Qi:** investigation (equal), supervision (equal), writing – review and editing (equal). **Ao Li:** investigation (equal), supervision (equal), writing – review and editing (equal). **Qiujun Wang:** data curation (equal), investigation (equal), supervision (equal), writing – review and editing (equal). **Zengyao Liu:** investigation (equal), supervision (equal), writing – review and editing (equal). **Ruitao Wang:** conceptualization (equal), data curation (equal), funding acquisition (equal), project administration (equal), supervision (equal), writing – review and editing (equal).

## Ethics Statement

This retrospective study was carried out after approval from the Institutional Review Board of three centers (Harbin Medical University Cancer Hospital, the First Affiliated Hospital of Harbin Medical University, and the Second Affiliated Hospital of Harbin Medical University). Because of the retrospective nature of the research, the requirement for informed consent was waived (IRB No. KY2022‐10, KY2023‐264, KY2022‐291).

## Conflicts of Interest

The authors declare no conflicts of interest.

## Supporting information


Supporting Information S1.

## Data Availability

The LDCT imaging data in the current study are not publicly available for patient privacy purposes. However, if researchers wish to access our data solely for scientific research purposes and are willing to sign a data transfer agreement, the corresponding author can share the relevant data.
