# Supplementary material for: An Integrated Nomogram Combining Deep Learning and Radiomics for Predicting Malignancy of Pulmonary Nodules Using CT‐Derived Nodules and Adipose Tissue: A Multicenter Study
Source: Cancer Med. 2024 Nov 4;13(21):e70372. doi: 10.1002/cam4.70372 (PMC11533136; doi:10.1002/cam4.70372)
Supplement: Supplementary file 1 — Supporting Information S1. [file CAM4-13-e70372-s001.docx]

**Supplementary material online for**

**“An integrated nomogram combining deep learning and radiomics for predicting malignancy of pulmonary nodules using CT-derived nodules and adipose tissue: a multicenter study”**

**This supplementary material includes:**

**Supplementary A1:** Patient recruitment

**Supplementary A2:** The CT examinations

**Supplementary A3:** Imaging data processing

**Supplementary A4:** Feature extraction

**Supplementary A5:** Feature selection

**Supplementary A6:** Stratification analysis

**References for Supplementary Materia**

**Supplementary Figure S1:** Intranodular and perinodular region deep learning feature selection graph

**Supplementary Figure S2:** Adipose tissue radiomic feature selection graph

**Supplementary Figure S3:** Stratification analysis

**Supplementary Figure S4:** The overall flow chart of the process of extracting adipose tissue mask from CT image

**Supplementary Table S1:** The CT image acquisition parameters of the three centers

**Supplementary Table S2:** The indices of IPN deep learning feature selection

**Supplementary Table S3:** Baseline model analysis

**Supplementary Table S4:** The performance comparison of machine learning

**Supplementary Table S5:** The performance comparison of hyperparameter combination

**Supplementary Table S6:** Construction of DLRCN via logistic regression analysis **Supplementary Table S7:** NRI and IDI comparison with and without the adipose tissue

**Supplementary Table S8:** NRI and IDI comparison with and without the adipose tissue in subgroups

**Supplementary A1: Patient recruitment**

A total of 1098 patients came from Harbin Medical University Cancer Hospital, the First Affiliated Hospital of Harbin Medical University, and the Second Affiliated Hospital of Harbin Medical University, they were divided into three cohorts all of which are detailed below.

**Primary cohort (PC)**

The primary cohort consisted of 550 patients from Center 1 (Harbin Medical University Cancer Hospital) between December 2016 and June 2022.

**Internal test cohort (I-T)**

The internal test cohort included 158 patients from Center 1 (Harbin Medical University Cancer Hospital) between December 2016 and June 2022.

**External test cohort 1 (E-T1)**

Independent external test cohort 1 included 191 patients from Center 2 (the First Affiliated Hospital of Harbin Medical University) between January 2021 and February 2023.

**External test cohort 2 (E-T2)**

Independent external test cohort 2 included 199 patients from Center 3 (the Second Affiliated Hospital of Harbin Medical University) between January 2019 and February 2023.

**Supplementary A2: The CT examinations**

Enrolled patients in the three centers underwent similar scan setups. The LDCT scans, covering the entire lung region, were acquired during a breath-hold with the patient supine. The LDCT image acquisition parameters of the three centers are shown in Supplementary Table S1. LDCT images of the transverse plane were retrieved from the picture archiving and communication system (PACS) and used in this study.

The diagnosis was performed with a standardized dynamic window adjustment procedure on window-adjustable PACS workstations. LDCT scans were independently reviewed by two chest radiologists with 10 (reader 1) and 5 (reader 2) years of experience, who were blinded to the pathologic diagnosis and medical history of the patients. The following subjective LDCT findings were noted: (a) the location of the pulmonary nodules (left upper lobe, left lower lobe, right upper lobe, right middle lobe, or right lower lobe); (b) the size of the lesion (the average of the major diameter and vertical short diameter measured on the section where the nodule appeared the largest in the lung window); (c) the presence of a spiculated sign (yes or no); (d) the lesion shape (round/oval or irregular); (e) the presence of a lobulated shape (yes or no). Lobulated shape was defined if an abrupt bulging of the contour of the lesion was seen. Spiculated sign was defined if thicker strands were seen extending from the nodule margin into the lung parenchyma without reaching the pleural surface^[[1]](#endnote-2)^.

**Supplementary A3: Imaging data processing**

**Image standardization**

To mitigate data variability across multicenter cohorts and facilitate the computation of quantitative radiological attributes, a procedure for image normalization was executed^[[2]](#endnote-3)^. This procedure transformed the initial images into a more standardized format. The adopted approach comprised a three-step image standardization process: Firstly, the images were transformed into a standard input format, featuring an intensity range spanning from -1024 to 1024 Hounsfield Units (HU), and adhering to a consistent chest window level (with a window level [WL] of -600) and window width [WW] of 1600). Subsequently, bilinear interpolation was applied to ensure uniform image proportions, resulting in an image size of 224×224 pixels. Finally, Z-scores were used to normalize the image^[[3]](#endnote-4)^.

**Intranodular and perinodular region (IPN)**

The region of interest (ROI) corresponding to the intranodular region was manually delineated by reader1. These delineations were performed on the slice of LDCT images featuring the largest nodule and were executed using 3D Slicer version 4.10.2 ([www.slicer.org](http://www.slicer.org/)). A binary mask representing the ROI was then generated for practical use. After that, we employed a dilation algorithm to create the 5mm perinodular mask. The IPN mask was created by combining these two masks. It is noteworthy that these operations were all performed on the original 512 × 512 pixels CT images.

After generating the IPN mask, which was a binary representation with values of 0 and 1, it was multiplied with the original image to isolate the IPN. During this process, the image size remained 512 × 512 pixels. Following this, a 128 × 128 pixels image centered on the nodule was cropped from this image. This was achieved by shifting 64 pixels to the left and upwards and 64 pixels to the right and downwards from the coordinates denoting the nodule's center. This method ensured that the center of the nodule corresponded precisely to the center of the cropped image, preserving all IPN information. Finally, the image underwent standardization procedures.

**Adipose tissue region**

The extraction process of the adipose tissue mask from LDCT images was detailed in Supplementary Figure S4. To obtain the region corresponding to adipose tissue, we utilized Image J to set a HU threshold ranging from -200 to -40 in the LDCT image (as shown in Supplementary Figure S4, A), resulting in the delineation of the red region, which encompassed all adipose tissue in the LDCT image.

Subsequently, as depicted in (Supplementary Figure S4, B), we manually traced this region with precision. This meticulous outlining process involved marking the region of adipose tissue, as illustrated in (Supplementary Figure S4, C).

Following this, we intersected the hand-drawn adipose tissue region with the previously obtained total LDCT adipose tissue region, yielding the final adipose tissue region. This intersection operation helped eliminate errors or misaligned sections, resulting in more precise adipose tissue regions and the subsequent generation of the corresponding mask (as shown in Supplementary Figure S4, D).

**Supplementary A4: Feature extraction**

**Deep learning feature extraction**

For deep learning feature extraction, we first initialized BiVGG using pre-trained weights from ImageNet, which contained more than 1,200,000 natural images belonging to 1,000 categories, and then fine-tuned the model^[[4]](#endnote-5)^.

Following the initialization of pre-training weights from ImageNet, a two-step fine-tuning training process was applied to the BiVGG. Specifically, all layers except the final one was initialized with pre-trained weights. In the fine-tuning phase, the training dataset comprised thousands of 224×224 images extracted from low-dose computed tomography (LDCT) of the primary cohort.

The mean square error served as the loss function, and the Adam optimizer was utilized to optimize the BiVGG. Each batch contained 32 images. In the initial step of fine-tuning, the convolutional layer remained unchanged, and only the final layer was trained with a relatively small initial learning rate (1e-5). In the subsequent step, all layers were trained, and the initial learning rate was further reduced to one-tenth of the first step's rate^[[5]](#endnote-6)^.

L1-L2 regularization was applied to the final layer to control overfitting and enhance the representation of the convolutional layer^[[6]](#endnote-7)^. Validation performance was assessed using a five-fold cross-validation approach. Learning rate scheduling utilized a step planning strategy with a gamma value of 0.9 and steps of 5. The early stopping patience value was set to 10 epochs.

PyTorch library ([https://www.pytorch.org](https://www.pytorch.org/)) were employed for the implementation of the BiVGG.

**Hand-crafted feature extraction**

Following the execution of IPN deep learning feature extraction, we proceeded to formulate 11 hand-crafted radiomic approaches. These hand-crafted approaches were designed to bestow radiomic nomenclature upon the deep learning features. The 11 hand-crafted radiomic approaches were delineated as follows.

1. H_uniformity:

$$uniformity=\sum_{i=1}^{N_{l}} {P(i)}^{2}$$

where $P$ is the histogram of region of interest (ROI) divided by $N_{l}$ intensity levels.

1. H_energy:

$$energy=\sum_{i}^{N} {X(i)}^{2}$$

where $X$ is the ROI with $N$ pixels.

1. H_skewness:

$$skewness=\frac{\frac{1}{N}\sum_{i=1}^{N} \left( X\left( i \right)-\bar{X} \right)^{3}}{\left( \sqrt{\frac{1}{N}\sum_{i=1}^{N} \left( X\left( i \right)-\bar{X} \right)^{2}} \right)^{3}}$$

where $X$ is the ROI with $N$ pixels, $\bar{X}$ is the mean of $X$.

1. H_root_mean_square:

$$root\_mean\_square=\sqrt{\frac{\sum_{i}^{N} X\left( i \right)^{2}}{N}}$$

where $X$ is the ROI with $N$ pixels.

1. H_variance:

$$variance=\frac{1}{N-1}\sum_{i=1}^{N} \left( X\left( i \right)-\bar{X} \right)^{2}$$

where $X$ is the ROI with $N$ pixels, $\bar{X}$ is the mean of $X$.

1. H_minimum: the minimum intensity value of the ROI.
2. H_maximum: the maximum intensity value of the ROI.
3. H_range: the range of intensity values of the ROI.
4. GLCM_dissimilarity:

$$dissimilarity=\sum_{i=1}^{N_{g}} \sum_{j=1}^{N_{g}} \left| i-j \right|P(i,j)$$

where $P(i,j)$ is the $(i,j)$th entry in the GLCM and $N_{g}$ the number of intensity levels in the ROI.

1. GLCM_homogeneity:

$$homogeneity=\sum_{i=1}^{N_{g}} \sum_{j=1}^{N_{g}} \frac{P(i,j)}{1+\left| i-j \right|}$$

where $P(i,j)$ is the $(i,j)$th entry in the GLCM and $N_{g}$ the number of intensity levels in the ROI.

1. GLCM_cluster_tendency:

$$cluster\_tendency=\sum_{i=1}^{N_{g}} \sum_{j=1}^{N_{g}} \left[ i+j-\mu_{x}(i)-\mu_{y}(j) \right]^{2}P(i,j)$$

where $P(i,j)$ is the $(i,j)$th entry in the GLCM, $N_{g}$ the number of intensity levels in the image, $\mu_{x}$ the mean of $p_{x}$, $\mu_{y}$ the mean of $p_{y}$.

**Supplementary A5: Feature selection**

**IPN deep learning feature selection**

For the IPN deep learning features, a four-step feature selection process was implemented. For each patient's nodule image, 512 feature maps with 7×7 pixels were extracted, and the dimensional representation for multiple patients was denoted as (P, C, H, W), where P represents the number of patients, C represents the number of channels, and H and W represent the height and width of the images. Since the training set comprised 440 patients, the initial dimensions for feature selection were (440×512×7×7). Firstly, the H and W dimensions of the feature maps were flattened to obtain (440×512×49). Secondly, Spearman correlation coefficients were calculated between the 512 features, indicating the correlations between the feature maps. Feature map groups with correlations greater than 0.6 were identified, and the feature maps with higher index were removed. After this step, 108 feature maps were selected, resulting in dimensions of (440×108×7×7). For each of these feature maps, 11 radiomic features were then extracted, providing the IPN deep learning features with radiomic nomenclature. The resulting dimensions were (440×108×11), or (440×1188). Subsequently, LASSO regression was applied to obtain the final selected IPN deep learning features. The "Take it all" method was employed during this LASSO regression step^[[7]](#endnote-8)^. Ultimately yielding 132 IPN deep learning selected features.

**Adipose tissue radiomic feature selection**

For the selection of adipose tissue radiomic features, out of the 1010 features extracted from radiomic method, LASSO regression was employed to select 10 features. These 10 features were then combined with clinical information, and both univariate and multivariate correlation analyses were conducted. Ultimately, only 2 adipose tissue radiomic features were identified as independent predictors.

**Supplementary A6: Stratification analysis**

Considering the possible confounding factors in the results, the subgroups of age, sex, body mass index (BMI), slice thickness, and CT system version were stratified.

**1. Stratified analysis on age:** Patients were divided into two subgroups: age < 55 and age >= 55 with AUC of 0.984 and 0.958.

**2. Stratified analysis on sex:** Patients were divided into two subgroups: female and male with AUC of 0.963 and 0.975.

**3. Stratified analysis on BMI:** Patients were divided into two subgroups: BMI < 24 and age >= 24 with AUC of 0.977 and 0.964.

**4. Stratified analysis on slice thickness:** Patients were divided into two subgroups: slice thickness < 5 and slice thickness >= 5 with AUC of 0.970 and 0.968.

**5. Stratified analysis on CT system version:** Patients were divided into three subgroups: GE medical systems, Philips and SIEMENS with AUC of 0.948, 0.937 and 0.979.

The results of the hierarchical analysis were shown in Supplementary Figure S3.

**Supplementary references**

**
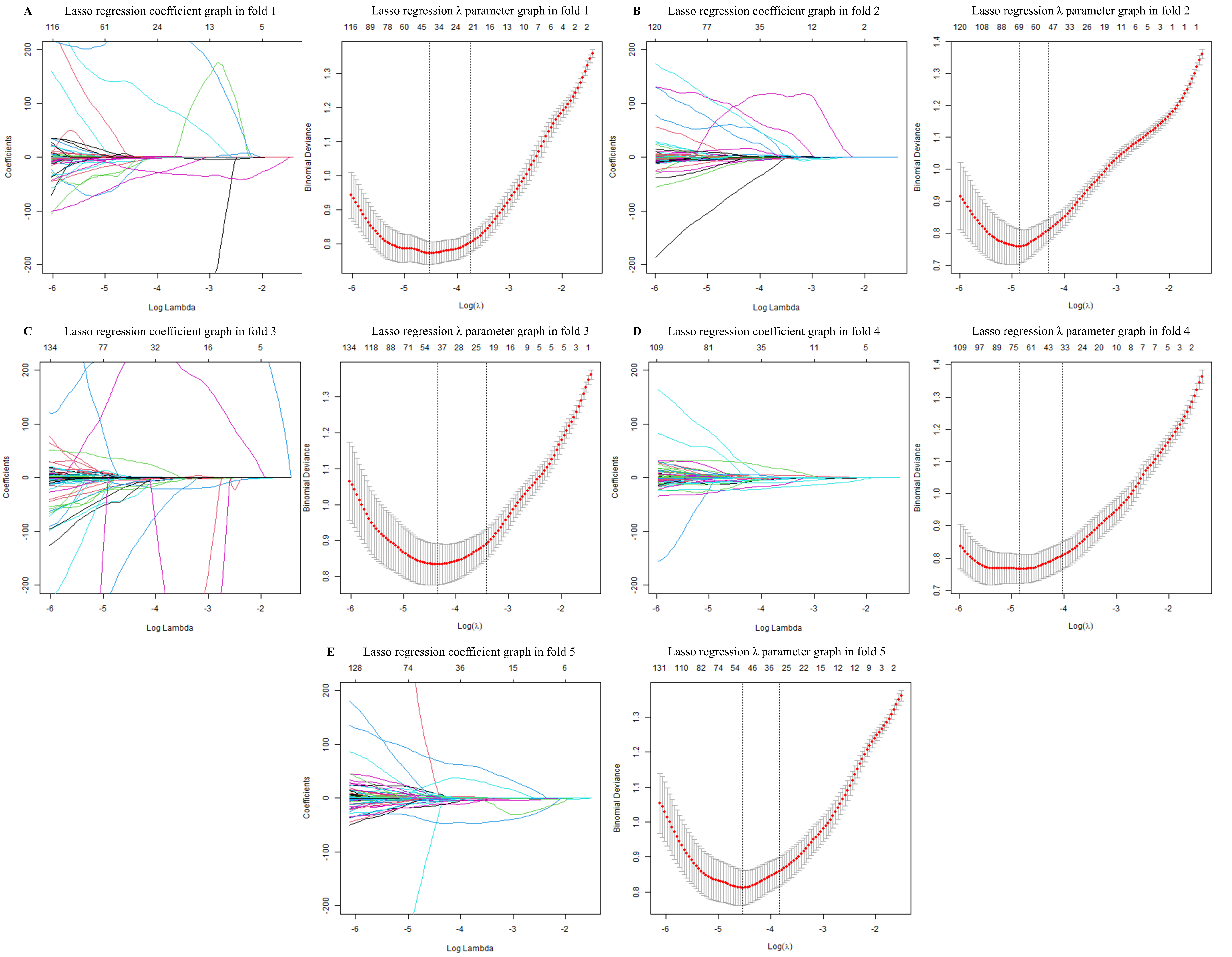
**

**Supplementary Figure S1. Intranodular and perinodular region deep learning feature selection graph.**

(A) Log λ = -2.7, feature count = 17; (B) Log λ = -2.8, feature count = 53; (C) Log λ = -2.6, feature count = 21; (D) Log λ = -2.4, feature count = 31; (E) Log λ = -2.2, feature count = 30.


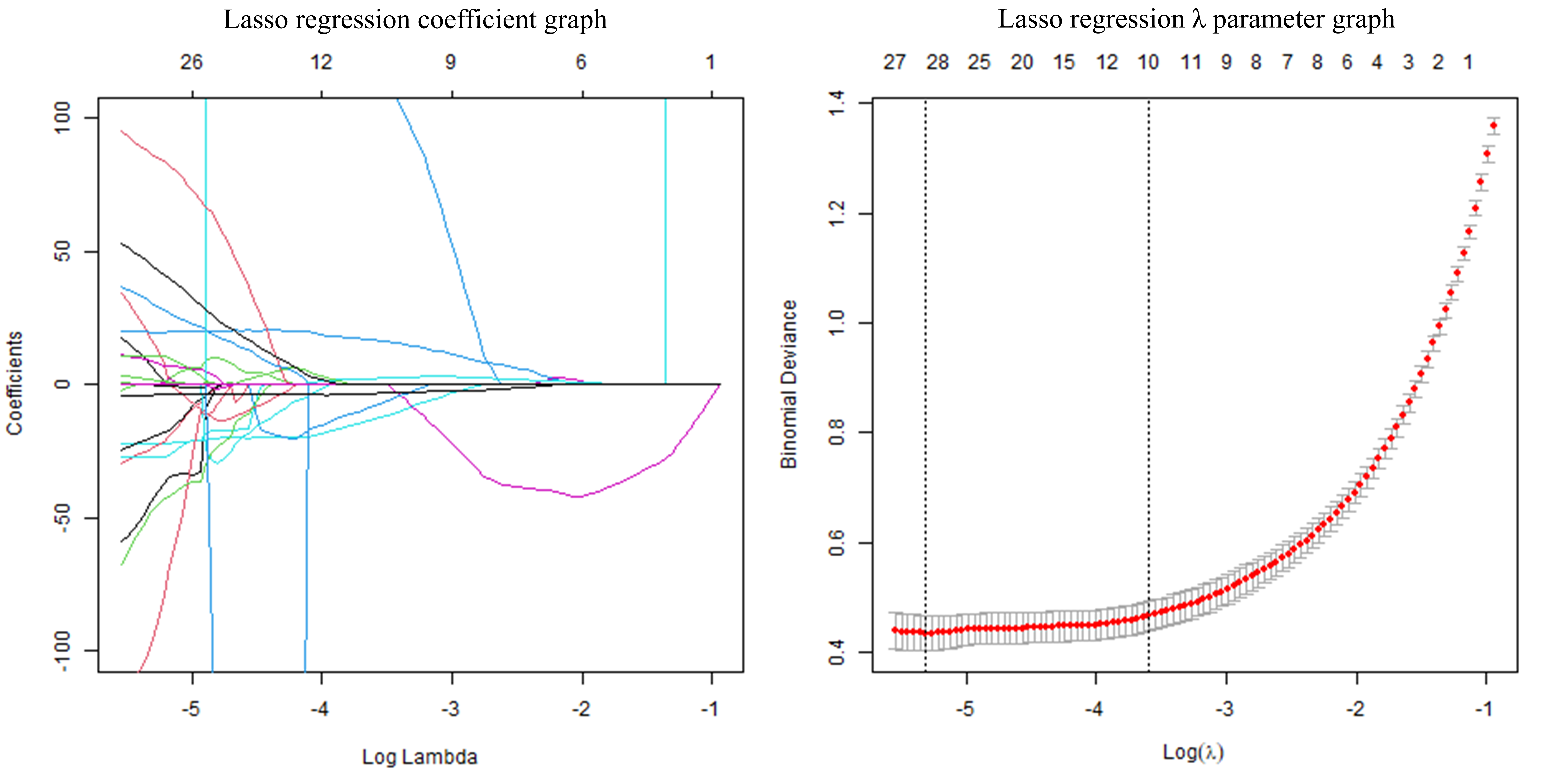


**Supplementary Figure S2. Adipose tissue radiomic feature selection graph.**

Log λ = -2.9, feature count = 10.


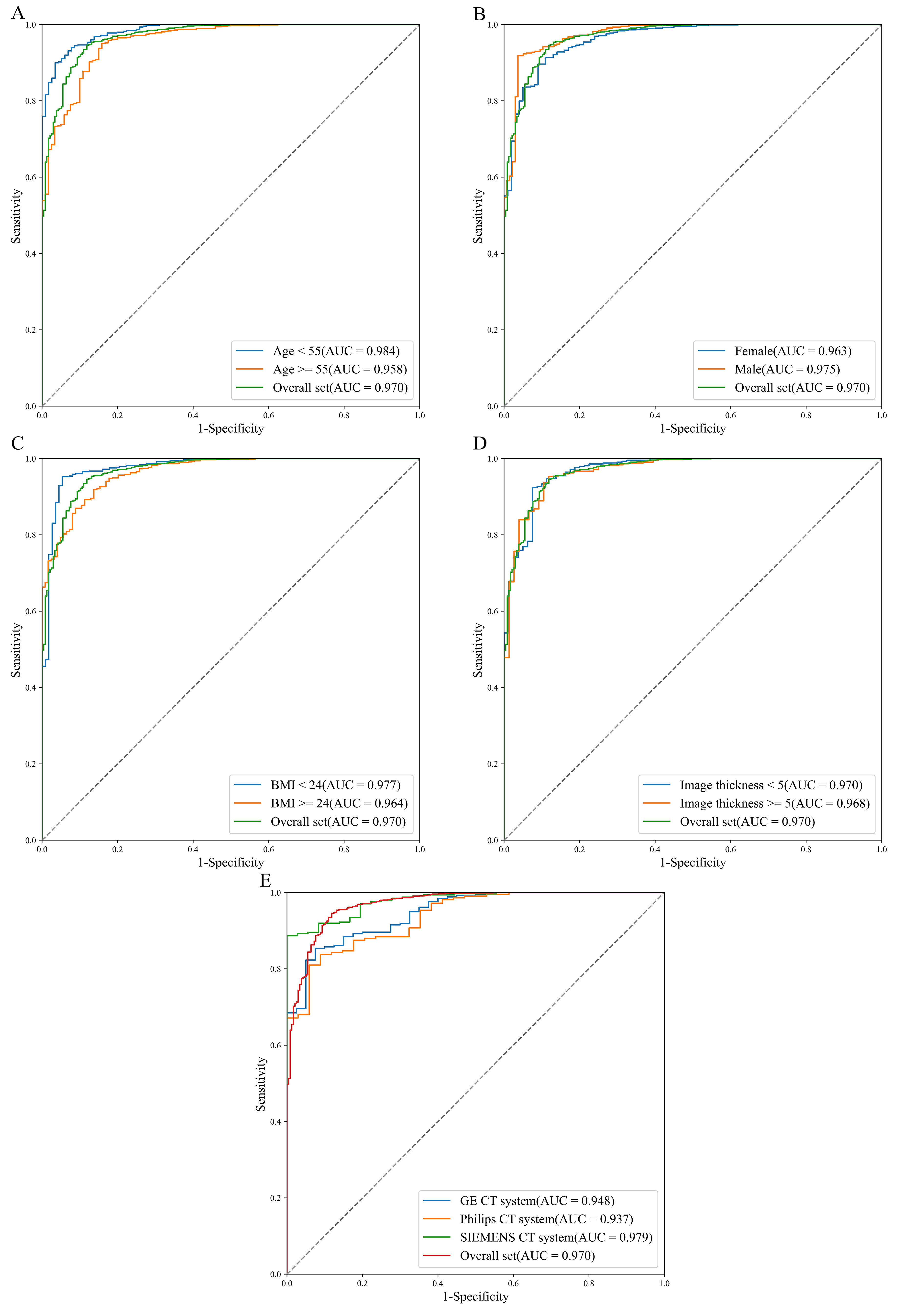


**Supplementary Figure S3. Stratification analysis.**

Images show each stratified area under the curve (AUC) compared by (A) Age; (B) Sex; (C) Body mass index (BMI); (D) Slice thickness; (E) Computed tomography (CT) system version.


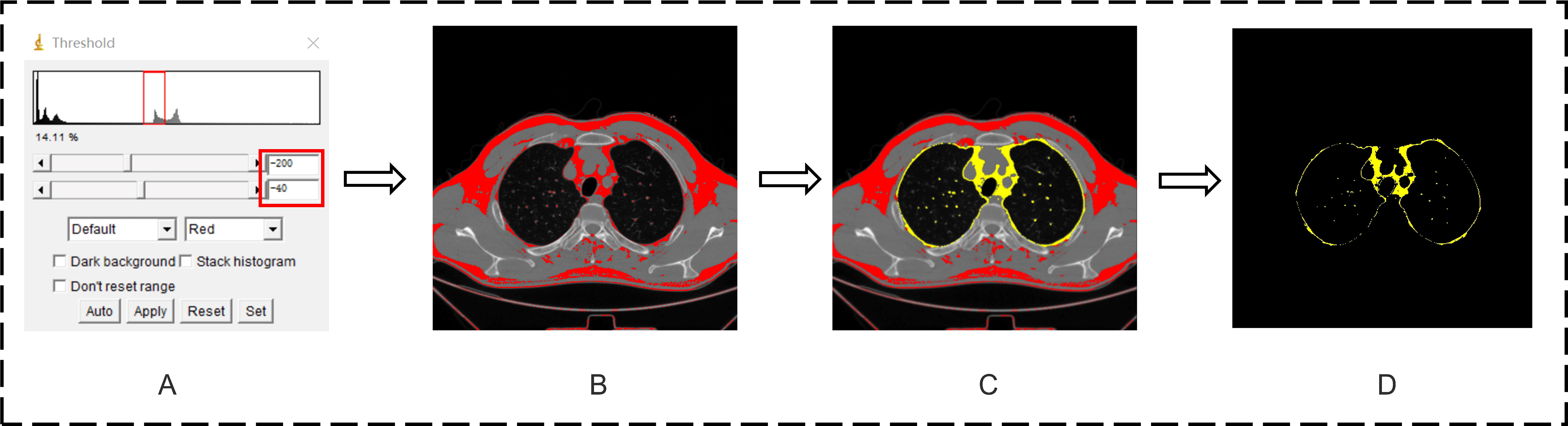


**Supplementary Figure S4. The overall flow chart of the process of extracting adipose tissue mask from CT image.**

(A) The threshold adjustment using Image J; (B) All the adipose tissue present in the image; (C) Intersection operation, resulting in the adipose tissue region; (D) The generated mask for the adipose tissue region.

| **Supplementary Table S1. The CT image acquisition parameters of the three centers.** | | | | |
| --- | --- | --- | --- | --- |
| **Parameters** | | **Centers** | | |
|  |  | Center 1  Harbin Medical University Cancer Hospital | Center 2  The First Affiliated Hospital, Harbin Medical University | Center 3  The Second Affiliated Hospital, Harbin Medical University |
| CT system  information | CT system | 256-slice spiral CT (SOMATOM Definition Flash, Siemens Healthineers, Germany) or a 64-slice spiral CT (Brilliance 64, PHILIPS, Netherlands) or a 16-slice spiral CT (BrightSpeed, GE Medical Systems, USA) | 256-slice spiral CT (Brilliance iCT, ROYAL PHILIPS, Netherlands) or a 64-slice spiral CT (Brilliance 16, PHILIPS, Netherlands) | 256-slice spiral CT (Brilliance iCT, ROYAL PHILIPS, Netherlands) or a 64-slice spiral CT (Discovery CT750 HD scanner, GE Medical Systems, USA) or a 16-slice spiral CT (SOMATON Sensation 10, Siemens Healthineers, Germany) |
| CT scan parameters | Tube voltage | 120 kVp | 120 kVp | 120 kVp |
|  | Tube current | 200-400 mA | 200–400 mA | 150-300 mA |
|  | Rotation time | 0.5 s | 0.5 s | 0.5 s |
|  | Detector collimation | 128×0.625 mm or 64×0.625 mm or 32×0.6 mm | 128×0.625 mm | 128×0.625 mm or 64×0.625 mm or 32×0.6 mm |
|  | Arterial phase CT | 35 s after injection | 28 s after injection | 25-35 s after injection |
|  | Venous phase CT | 60 s after injection | 60 s after injection | 55-65 s after injection |
| CT image information | With unenhanced CT | Yes | Yes | Yes |
|  | Image matrix | 512×512 | 512×512 | 512×512 |
|  | Field of view | 400×400 mm or 500×500 mm | 400×400 mm | 400×400 mm or 500×500 mm |
|  | Reconstruction section thickness | 1.25 mm for enhanced CT  5 mm for both enhanced and unenhanced CT | 5 mm for both enhanced and unenhanced CT | 1.25 mm for enhanced CT  5 mm for both enhanced and unenhanced CT  0.625 mm for enhanced CT |
| NOTICE: CT, computed tomography. | | | | |

**Supplementary Table S2. The indices of IPN deep learning feature selection.**

| **Folds** | **Spearman** | | **LASSO** | |
| --- | --- | --- | --- | --- |
|  | Indices in feature maps | “Take it all” indices | Indices in features | “Take it all” indices |
| Fold1 | [0, 9, 10, 15, 21, 24, 30, 49, 50, 73, 78, 140, 180, 201, 294, 431, 442] | [0, 1, 2, 3, 4, 5, 6, 7, 8, 9, 10, 12, 13, 14, 15, 16, 18, 20, 21, 22, 23, 24, 25, 26, 27, 28, 29, 30, 31, 32, 34, 35, 38, 39, 41, 49, 50, 51, 52, 53, 55, 62, 65, 66, 67, 68, 71, 73, 75, 78, 80, 84, 85, 86, 87, 103, 110, 111, 112, 115, 116, 117, 123, 131, 140, 141, 144, 148, 155, 156, 163, 165, 166, 171, 178, 179, 180, 187, 195, 201, 209, 228, 239, 250, 258, 267, 271, 294, 314, 319, 325, 347, 359, 365, 366, 393, 410, 414, 429, 431, 435, 442, 443, 484, 486, 495, 506, 510] | [175, 176, 221, 235, 283, 370, 397, 404, 461, 637, 641, 880, 931, 947, 1003, 1033, 1047] | [0, 44, 75, 97, 109, 112, 161, 165, 170, 173, 174, 214, 219, 220, 231, 233, 262, 266, 273, 280, 281, 283, 299, 319, 321, 347, 349, 361, 365, 368, 371, 379, 387, 390, 395, 402, 417, 428, 456, 459, 462, 483, 492, 493, 515, 517, 530, 536, 537, 541, 545, 548, 559, 570, 602, 607, 613, 614, 624, 625, 633, 635, 638, 639, 647, 649, 659, 690, 702, 725, 736, 742, 745, 750, 756, 777, 779, 789, 814, 819, 823, 830, 841, 847, 853, 856, 858, 863, 878, 887, 889, 897, 900, 921, 929, 932, 933, 934, 935, 941, 945, 948, 954, 956, 958, 968, 973, 976, 987, 1001, 1005, 1019, 1031, 1032, 1034, 1039, 1043, 1044, 1045, 1062, 1080, 1088, 1097, 1117, 1120, 1128, 1143, 1155, 1163, 1171, 1183, 1185] |
| Fold2 | [0, 1, 2, 4, 5, 6, 12, 20, 26, 28, 29, 30, 31, 32, 35, 38, 39, 53, 55, 65, 67, 68, 71, 75, 84, 85, 87, 103, 110, 112, 117, 144, 166, 171, 178, 187, 228, 239, 250, 258, 271, 314, 319, 359, 365, 410, 429, 486, 495] |  | [77, 99, 114, 172, 216, 233, 268, 275, 285, 301, 349, 363, 381, 392, 419, 458, 495, 517, 538, 539, 572, 604, 616, 626, 649, 744, 752, 821, 825, 832, 843, 858, 860, 865, 902, 923, 936, 943, 956, 958, 975, 978, 1021, 1041, 1045, 1046, 1082, 1119, 1122, 1130, 1145, 1157, 1185] |  |
| Fold3 | [0, 9, 10, 15, 24, 27, 35, 49, 73, 78, 209, 393, 431, 435] |  | [2, 111, 167, 176, 283, 321, 367, 370, 397, 430, 752, 889, 923, 931, 935, 1007, 1033, 1047, 1064, 1099, 1119] |  |
| Fold4 | [0, 1, 2, 3, 4, 5, 7, 8, 10, 12, 13, 14, 15, 18, 22, 23, 24, 25, 28, 30, 32, 34, 41, 62, 66, 80, 86, 103, 111, 115, 116, 123, 131, 141, 156, 163, 165, 179, 195, 228, 325, 347, 414, 429, 443, 484, 506] |  | [221, 222, 282, 285, 351, 389, 397, 485, 532, 538, 561, 609, 635, 640, 651, 661, 692, 727, 738, 747, 791, 849, 855, 934, 937, 950, 958, 970, 989, 1034, 1090] |  |
| Fold5 | [0, 9, 10, 15, 16, 21, 27, 30, 35, 51, 52, 148, 155, 267, 366, 510] |  | [46, 163, 175, 176, 235, 264, 323, 370, 373, 464, 494, 519, 543, 547, 550, 615, 627, 704, 758, 779, 781, 816, 891, 899, 960, 1033, 1036, 1165, 1173, 1187] |  |

NOTICE: IPN, intranodular and perinodular region.

**Supplementary Table S3. Baseline model analysis.**

| **Cohorts** | **Fold1 AUC** | **Fold2 AUC** | **Fold3 AUC** | **Fold4 AUC** | **Fold5 AUC** | **Mean AUC^*^** |
| --- | --- | --- | --- | --- | --- | --- |
| Training cohort |  |  |  |  |  |  |
| BiVGG | 0.917 | 0.902 | 0.911 | 0.895 | 0.885 | 0.902[0.889,0.915] |
| VGG16 | 0.911 | 0.890 | 0.891 | 0.897 | 0.911 | 0.900[0.899,0.911] |
| VGG19 | 0.907 | 0.873 | 0.837 | 0.831 | 0.912 | 0.873[0.834,0.912] |
| Resnet34 | 0.908 | 0.885 | 0.817 | 0.890 | 0.907 | 0.881[0.843,0.920] |
| Resnet50 | 0.776 | 0.862 | 0.745 | 0.792 | 0.768 | 0.789[0.743,0.834] |
| Desnet121 | 0.895 | 0.786 | 0.857 | 0.816 | 0.840 | 0.835[0.799,0.870] |
| Desnet161 | 0.768 | 0.793 | 0.901 | 0.858 | 0.857 | 0.835[0.780,0.891] |
| Validation cohort |  |  |  |  |  |  |
| BiVGG | 0.862 | 0.884 | 0.811 | 0.798 | 0.831 | 0.837[0.800,0.874] |
| VGG16 | 0.811 | 0.875 | 0.658 | 0.845 | 0.853 | 0.808[0.719,0.898] |
| VGG19 | 0.873 | 0.871 | 0.672 | 0.798 | 0.843 | 0.811[0.725,0.898] |
| Resnet34 | 0.772 | 0.701 | 0.684 | 0.818 | 0.858 | 0.767[0.689,0.844] |
| Resnet50 | 0.703 | 0.778 | 0.645 | 0.632 | 0.713 | 0.694[0.634,0.754] |
| Desnet121 | 0.780 | 0.778 | 0.716 | 0.702 | 0.758 | 0.747[0.710,0.784] |
| Desnet161 | 0.743 | 0.780 | 0.709 | 0.707 | 0.733 | 0.734[0.704,0.765] |
| Internal test cohort |  |  |  |  |  |  |
| BiVGG | 0.845 | 0.812 | 0.834 | 0.818 | 0.853 | 0.832[0.814,0.850] |
| VGG16 | 0.812 | 0.818 | 0.769 | 0.833 | 0.860 | 0.818[0.784,0.853] |
| VGG19 | 0.840 | 0.801 | 0.713 | 0.732 | 0.827 | 0.783[0.724,0.842] |
| Resnet34 | 0.676 | 0.677 | 0.667 | 0.707 | 0.694 | 0.685[0.668,0.701] |
| Resnet50 | 0.678 | 0.704 | 0.673 | 0.658 | 0.661 | 0.675[0.656,0.694] |
| Desnet121 | 0.745 | 0.745 | 0.723 | 0.742 | 0.716 | 0.734[0.720,0.749] |
| Desnet161 | 0.698 | 0.746 | 0.760 | 0.752 | 0.785 | 0.748[0.728,0.768] |
| External test cohort 1 |  |  |  |  |  |  |
| BiVGG | 0.818 | 0.806 | 0.828 | 0.832 | 0.825 | 0.822[0.811,0.832] |
| VGG16 | 0.837 | 0.825 | 0.808 | 0.824 | 0.793 | 0.817[0.800,0.835] |
| VGG19 | 0.859 | 0.824 | 0.788 | 0.800 | 0.813 | 0.817[0.789,0.845] |
| Resnet34 | 0.787 | 0.752 | 0.755 | 0.777 | 0.750 | 0.764[0.747,0.781] |
| Resnet50 | 0.733 | 0.725 | 0.729 | 0.732 | 0.766 | 0.737[0.721,0.754] |
| Desnet121 | 0.793 | 0.785 | 0.788 | 0.788 | 0.775 | 0.786[0.779,0.793] |
| Desnet161 | 0.758 | 0.805 | 0.810 | 0.811 | 0.829 | 0.803[0.786,0.819] |
| External test cohort 2 |  |  |  |  |  |  |
| BiVGG | 0.842 | 0.857 | 0.877 | 0.829 | 0.810 | 0.843[0.817,0.869] |
| VGG16 | 0.835 | 0.844 | 0.841 | 0.846 | 0.820 | 0.837[0.826,0.848] |
| VGG19 | 0.863 | 0.837 | 0.826 | 0.827 | 0.811 | 0.833[0.814,0.853] |
| Resnet34 | 0.803 | 0.804 | 0.796 | 0.823 | 0.777 | 0.800[0.783,0.817] |
| Resnet50 | 0.796 | 0.806 | 0.765 | 0.791 | 0.786 | 0.789[0.774,0.805] |
| Desnet121 | 0.769 | 0.795 | 0.814 | 0.776 | 0.790 | 0.790[0.771,0.808] |
| Desnet161 | 0.768 | 0.823 | 0.759 | 0.797 | 0.833 | 0.796[0.776,0.817] |
| NOTICE: AUC, area under the curve.  * Data in brackets are 95% confidence intervals. | | | | | | |

**Supplementary Table S4. The performance comparison of machine learning.**

| **Cohorts** | **AUC^*^** | **ACC^*^** | **SEN^*^** | **SPE^*^** |
| --- | --- | --- | --- | --- |
| Training cohort |  |  |  |  |
| LR | 0.934[0.927,0.941] | 0.838[0.827,0.849] | 0.799[0.787,0.811] | 0.892[0.878,0.906] |
| SVM | 0.953[0.946,0.960] | 0.890[0.883,0.897] | 0.909[0.887,0.931] | 0.863[0.824,0.902] |
| RF | 0.976[0.973,0.979] | 0.923[0.917,0.929] | 0.920[0.902,0.938] | 0.927[0.911,0.943] |
| Validation cohort |  |  |  |  |
| LR | 0.874[0.867,0.881] | 0.778[0.773,0.783] | 0.769[0.756,0.782] | 0.791[0.775,0.807] |
| SVM | 0.885[0.873,0.897] | 0.816[0.800,0.832] | 0.856[0.831,0.881] | 0.761[0.711,0.811] |
| RF | 0.899[0.894,0.904] | 0.796[0.779,0.813] | 0.806[0.769,0.843] | 0.783[0.740,0.826] |
| Internal test cohort |  |  |  |  |
| LR | 0.767[0.737,0.797] | 0.696[0.680,0.712] | 0.684[0.663,0.705] | 0.752[0.692,0.812] |
| SVM | 0.772[0.755,0.789] | 0.743[0.723,0.763] | 0.769[0.725,0.813] | 0.628[0.534,0.722] |
| RF | 0.807[0.787,0.827] | 0.729[0.706,0.752] | 0.733[0.700,0.766] | 0.710[0.642,0.778] |
| External test cohort 1 |  |  |  |  |
| LR | 0.801[0.780,0.822] | 0.711[0.702,0.720] | 0.701[0.684,0.718] | 0.750[0.686,0.814] |
| SVM | 0.791[0.765,0.817] | 0.735[0.719,0.751] | 0.760[0.731,0.789] | 0.640[0.534,0.746] |
| RF | 0.807[0.794,0.820] | 0.714[0.695,0.733] | 0.694[0.673,0.715] | 0.790[0.761,0.819] |
| External test cohort 2 |  |  |  |  |
| LR | 0.763[0.748,0.778] | 0.658[0.646,0.670] | 0.614[0.591,0.637] | 0.829[0.783,0.875] |
| SVM | 0.759[0.739,0.779] | 0.713[0.678,0.748] | 0.728[0.676,0.780] | 0.654[0.588,0.720] |
| RF | 0.812[0.804,0.820] | 0.716[0.699,0.733] | 0.695[0.667,0.723] | 0.795[0.741,0.849] |
| NOTICE: AUC, area under the curve; ACC, accuracy; SEN, sensitivity; SPE, specificity; SVM, support vector machine; LR, logistic regression; RF, random forest.  * Data in brackets are 95% confidence intervals. | | | | |

**Supplementary Table S5. The performance comparison of hyperparameter combination.**

| **LR** | | | | **SVM** | | | | **RF** | | | | |
| --- | --- | --- | --- | --- | --- | --- | --- | --- | --- | --- | --- | --- |
| Hyperparameter | | Training AUC | Validation AUC | Hyperparameter | | Training AUC | Validation AUC | Hyperparameter | | | Training AUC | Validation AUC |
| C | Penalty |  |  | C | Kernel |  |  | N estimators | Min samples leaf | Max depth |  |  |
| 0.001 | L1 | 0.850 | 0.801 | 0.001 | Linear | 0.873 | 0.793 | 25 | 11 | 4 | 0.978 | 0.891 |
| 0.001 | L2 | 0.847 | 0.796 | 0.001 | RBF | 0.787 | 0.756 | 25 | 11 | 5 | 0.985 | 0.903 |
| 0.01 | L1 | 0.868 | 0.789 | 0.01 | Linear | 0.893 | 0.817 | 25 | 11 | 6 | 0.988 | 0.906 |
| 0.01 | L2 | 0.886 | 0.805 | 0.01 | RBF | 0.788 | 0.758 | 25 | 12 | 4 | 0.976 | 0.897 |
| 0.1 | L1 | 0.890 | 0.814 | 0.1 | Linear | 0.933 | 0.858 | 25 | 12 | 5 | 0.983 | 0.905 |
| 0.1 | L2 | 0.922 | 0.843 | 0.1 | RBF | 0.803 | 0.774 | 25 | 12 | 6 | 0.986 | 0.902 |
| 1 | L1 | 0.949 | 0.863 | 1 | Linear | 0.952 | 0.859* | 25 | 13 | 4 | 0.976 | 0.900 |
| 1 | L2 | 0.945 | 0.863 | 1 | RBF | 0.843 | 0.808 | 25 | 13 | 5 | 0.982 | 0.904 |
| 10 | L1 | 0.970 | 0.843 | 10 | Linear | 0.880 | 0.841 | 25 | 13 | 6 | 0.984 | 0.907* |
| 10 | L2 | 0.948 | 0.873* | 10 | RBF | 0.853 | 0.839 | 50 | 11 | 4 | 0.973 | 0.893 |
| 100 | L1 | 0.997 | 0.861 | 100 | Linear | 0.842 | 0.821 | 50 | 11 | 5 | 0.983 | 0.898 |
| 100 | L2 | 0.943 | 0.861 | 100 | RBF | 0.833 | 0.813 | 50 | 11 | 6 | 0.987 | 0.900 |
|  |  |  |  |  |  |  |  | 50 | 12 | 4 | 0.972 | 0.896 |
|  |  |  |  |  |  |  |  | 50 | 12 | 5 | 0.982 | 0.900 |
|  |  |  |  |  |  |  |  | 50 | 12 | 6 | 0.986 | 0.904 |
|  |  |  |  |  |  |  |  | 50 | 13 | 4 | 0.973 | 0.892 |
|  |  |  |  |  |  |  |  | 50 | 13 | 5 | 0.982 | 0.900 |
|  |  |  |  |  |  |  |  | 50 | 13 | 6 | 0.984 | 0.900 |
|  |  |  |  |  |  |  |  | 75 | 11 | 4 | 0.974 | 0.894 |
|  |  |  |  |  |  |  |  | 75 | 11 | 5 | 0.984 | 0.901 |
|  |  |  |  |  |  |  |  | 75 | 11 | 6 | 0.989 | 0.904 |
|  |  |  |  |  |  |  |  | 75 | 12 | 4 | 0.973 | 0.895 |
|  |  |  |  |  |  |  |  | 75 | 12 | 5 | 0.983 | 0.902 |
|  |  |  |  |  |  |  |  | 75 | 12 | 6 | 0.987 | 0.905 |
|  |  |  |  |  |  |  |  | 75 | 13 | 4 | 0.974 | 0.895 |
|  |  |  |  |  |  |  |  | 75 | 13 | 5 | 0.982 | 0.901 |
|  |  |  |  |  |  |  |  | 75 | 13 | 6 | 0.985 | 0.901 |

NOTICE: AUC, area under the curve; LR, logistic regression; SVM, support vector machine; RF, random forest; RBF, radial basis function.

* Optimal combination of hyperparameter

**Supplementary Table S6. Construction of DLRCN via logistic regression analysis.**

| **Variables** | **β±SE** | ***P* value** |
| --- | --- | --- |
| Intercept | 7.018±5.860 | *P=*0.023 |
| Sex | -1.634±0.616 | *P=*0.008 |
| Age | 0.111±0.033 | *P=*0.049 |
| Lesion shape | 2.770±0.699 | *P<*0.001 |
| Demarcation | 1.187±0.622 | *P=*0.004 |
| IPN signature | 5.760±1.220 | *P<*0.001 |
| Adipose tissue signature 1 | -3.423±2.736 | *P=*0.044 |
| Adipose tissue signature 2 | -4.554±1.706 | *P*=0.032 |

NOTICE: β, the regression coefficients; SE, standard error.

**Supplementary Table S7. NRI and IDI comparison with and without the adipose tissue.**

| **Cohorts** | **Clinical model vs Clinical adipose model** | | | | **Clinical IPN model vs DLRCN** | | | |
| --- | --- | --- | --- | --- | --- | --- | --- | --- |
|  | NRI* | IDI^*^ | Likelihood ratio test statistic | Likelihood ratio test *P*-value | NRI* | IDI^*^ | Likelihood ratio test statistic | Likelihood ratio test *P*-value |
| Internal test cohort | 0.514  [0.120,0.909] | 0.027  [0.016,0.037] | 10.869 | *P* < 0.001 | 1.028  [0.695,1.361] | 0.137  [0.090,0.184] | 27.943 | *P* < 0.001 |
| External test cohort 1 | 0.960  [0.651,1.269] | 0.034  [0.020,0.048] | 11.344 | *P* < 0.001 | 0.633  [0.297,0.970] | 0.114  [0.071,0.157] | 56.324 | *P* < 0.001 |
| External test cohort 2 | 0.049  [0.025,0.143] | 0.054  [0.002,0.190] | 51.470 | *P* < 0.001 | 0.320  [0.022,0.618] | 0.088  [0.016,0.122] | 85.441 | *P* < 0.001 |
| NOTICE: DLRCN, deep learning radiomic clinical nomogram; IPN, intranodular and perinodular region; NRI, net reclassification improvement; IDI, integrated discrimination improvement.  * Data in brackets are 95% confidence intervals. | | | | | | | | |

**Supplementary Table S8. NRI and IDI comparison with and without the adipose tissue in subgroups.**

| **Cohorts** | **Clinical model vs Clinical adipose model** | | | | **Clinical IPN model vs DLRCN** | | | |
| --- | --- | --- | --- | --- | --- | --- | --- | --- |
|  | NRI* | IDI^*^ | Likelihood ratio test statistic | Likelihood ratio test *P*-value | NRI* | IDI^*^ | Likelihood ratio test statistic | Likelihood ratio test *P*-value |
| Solid |  |  |  |  |  |  |  |  |
| Internal test cohort | 1.478  [1.134,1.673] | 0.234  [0.162,0.306] | 19.188 | *P* < 0.001 | 1.477  [1.166,1.789] | 0.188  [0.124,0.252] | 16.801 | *P* < 0.001 |
| External test cohort 1 | 0.408  [0.092,0.907] | 0.044  [0.031,0.119] | 13.901 | *P* = 0.023 | 0.302  [0.193,0.798] | 0.076  [0.062,0.081] | 17.177 | *P* < 0.001 |
| External test cohort 2 | 0.647  [0.173,1.121] | 0.113  [0.023,0.203] | 20.871 | *P* < 0.001 | 0.105  [0.095,0.306] | 0.138  [0.061,0.215] | 18.277 | *P* < 0.001 |
| Subsolid |  |  |  |  |  |  |  |  |
| Internal test cohort | 0.867  [0.317,1.418] | 0.121  [0.024,0.218] | 16.067 | *P* = 0.004 | 0.764  [0.209,1.319] | 0.053  [0.016,0.151] | 18.607 | *P* < 0.001 |
| External test cohort 1 | 1.024  [0.617,1.431] | 0.165  [0.079,0.252] | 17.058 | *P* < 0.001 | 0.933  [0.522,1.344] | 0.138  [0.059,0.217] | 8.569 | *P* = 0.003 |
| External test cohort 2 | 0.091  [0.083,0.165] | 0.012  [0.002,0.029] | 31.941 | *P* < 0.001 | 0.182  [0.058,0.422] | 0.088  [0.021,0.157] | 35.351 | *P* < 0.001 |
| NOTICE: DLRCN, deep learning radiomic clinical nomogram; IPN, intranodular and perinodular region; NRI, net reclassification improvement; IDI, integrated discrimination improvement.  * Data in brackets are 95% confidence intervals. | | | | | | | | |

1. . Hansell DM, Bankier AA, MacMahon H, McLoud TC, Müller NL, Remy J. Fleischner Society: glossary of terms for thoracic imaging. Radiology. 2008 Mar;246(3):697-722. [↑](#endnote-ref-2)
2. . Russakovsky O, Deng J, Su H. ImageNet Large Scale Visual Recognition Challenge. Int J Comput Vis. 2015;115:211-252. [↑](#endnote-ref-3)
3. . Shalabi Luai A. Al, Zyad Shaaban, Basel Kasasbeh. Data Mining: A Preprocessing Engine. Journal of Computer Science. 2006;735-739. [↑](#endnote-ref-4)
4. . Russakovsky, Olga, Jia Deng, Hao Su, Jonathan Krause, Sanjeev Satheesh, Sean Ma, Zhiheng Huang, Andrej Karpathy, Aditya Khosla, Michael S. Bernstein, Alexander C. Berg, Li Fei-Fei. ImageNet Large Scale Visual Recognition Challenge. International Journal of Computer Vision. 2014;115:211-252. [↑](#endnote-ref-5)
5. . Käding C, Rodner E, Freytag A, Denzler J. Fine-Tuning Deep Neural Networks in Continuous Learning Scenarios. Computer Vision – ACCV 2016 Workshops. 2017; 588–605 [↑](#endnote-ref-6)
6. . Demir-Kavuk O, Kamada M, Akutsu T, Knapp EW. Prediction using step-wise L1, L2 regularization and feature selection for small data sets with large number of features. BMC Bioinformatics. 2011 Oct 25;12:412. [↑](#endnote-ref-7)
7. . Rokach L, Chizi B, Maimon O. A Methodology for Improving the Performance of Non-Ranker Feature Selection Filters. Int. J. Pattern Recognit. Artif. Intell. 2007 Aug 1;21:809-830. [↑](#endnote-ref-8)
